# Multicolor ultralong phosphorescence from perovskite-like octahedral α-AlF_3_

**DOI:** 10.1038/s41467-022-33540-1

**Published:** 2022-09-29

**Authors:** Peisheng Cao, Haoyue Zheng, Peng Wu

**Affiliations:** 1 College of Chemistry, Sichuan University, Chengdu, 610064 China; 2 Analytical & Testing Center, Sichuan University, Chengdu, 610064 China

**Keywords:** Materials chemistry, Materials for optics, Inorganic chemistry

## Abstract

Designing organic fluorescent and phosphorescent materials based on various core fluorophore has gained great attention, but it is unclear whether similar luminescent units exist for inorganic materials. Inspired by the BX_6_ octahedral structure of luminescent metal halide perovskites (MHP), here we propose that the BX_6_ octahedron may be a core structure for luminescent inorganic materials. In this regard, excitation-dependent color-tunable phosphorescence is discovered from α-AlF_3_ featuring AlF_6_ octahedron. Through further exploration of the BX_6_ unit by altering the dimension and changing the center metal (B) and ligand (X), luminescence from KAlF_4_, (NH_4_)_3_AlF_6_, AlCl_3_, Al(OH)_3_, Ga_2_O_3_, InCl_3_, and CdCl_2_ are also discovered. The phosphorescence of α-AlF_3_ can be ascribed to clusterization-triggered emission, i.e., weak through space interaction of the *n* electrons of F atoms bring close proximity in the AlF_6_ octahedra (inter/intra). These discoveries will deepen the understanding and contribute to further development of BX_6_ octahedron-based luminescent materials.

## Abstract (web summary)

Unravelling the origin of emission in luminescent inorganic materials is challenging. Here, the authors report that AlF_6_ octahedrons exhibit excitation-dependent color-tunable phosphorescence; structurally related compounds are also luminescent.

## Introduction

**Fig. 1:**
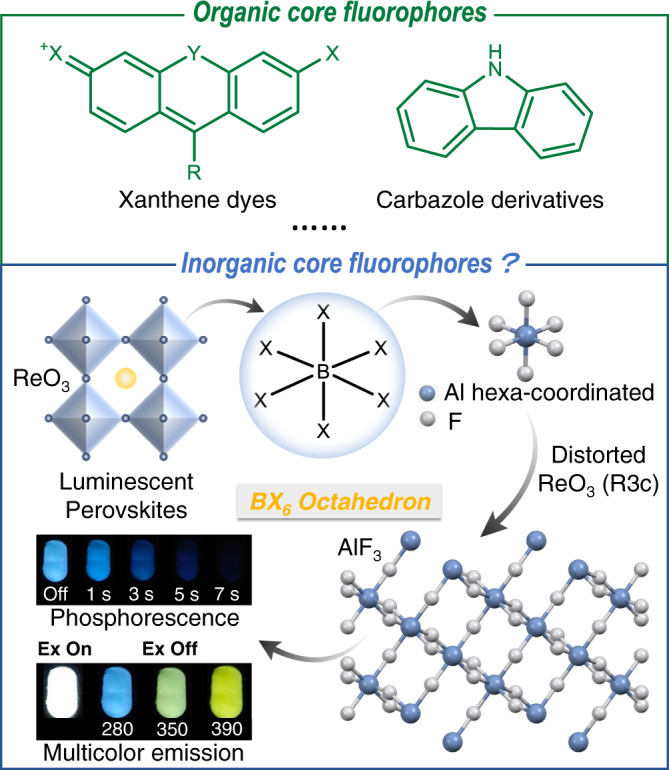
Schematic illustration of luminescent materials design. Inspired by the general luminescent material design based on organic core fluorophores and the widely investigated metal halide perovskites, here, we proposed BX_6_ octahedral unit as inorganic luminescent core structure.

Luminescent materials are indispensable for our daily life, especially in lighting, displaying, and imaging-related applications^1,2^. Therefore, luminescent materials design (either organic or inorganic) is of great importance and attracts great attention. It is widely accepted that luminescence from organic materials can be ascribed to their core structure in most cases (Fig. 1, together with the substituents), for example, fluorescent materials from xanthene^3–7^ and phosphorescent materials based on carbazole^8–11^. Such core structure endow organic fluorophores with great flexibility and processability. While for inorganic luminescent materials, although structurally diverse and mostly acting as host materials for doping of transition- or rare-earth metal ions (e.g., ZnS^12^ and SrAl_2_O_4_^13^), core structure as the light-emitting unit has seldom been reported and explored like organics. So, is there similar core structure for the luminescent inorganic materials?

All-inorganic metal halide perovskites (MHPs), a type of semiconductor materials with excellent photoelectric properties^14^, have been widely used in solar cells^15^, LED^16^, and thermoelectric modules^17^. The core structure of luminescent MHPs can be described as the BX_6_ octahedron (Fig. 1), which is constituted by the central cation (B, hexa-coordinated) and six halide ligands (X=Cl, Br, I). Normally, the BX_6_ octahedron is organized in an all-corner-sharing 3D network. Due to the adjustable octahedral connectivity, a series of lower dimensional metal halide-based luminescent perovskite derivatives have been reported^18^. On the other hand, the central cation and ligand halides could be altered, leading to tunable luminescence performance from 3D and lower dimension metal halides^19,20^. Therefore, the BX_6_ octahedron is an important structure for the luminescence of MHPs, but whether such unit can be generalized for other luminescent inorganic materials remains unexplored.

In this work, we find that inorganic materials constructed by the BX_6_ octahedron exhibit interesting long-lived room temperature phosphorescence (RTP), i.e., the BX_6_ octahedron may be regarded as a basic unit for the luminescent inorganic materials. For example, α-AlF_3_, the material constructed by AlF_6_ (the lightest BX_6_ octahedron) with 3D perovskite-like structure^21^, shows color-tunable RTP (up to 7 s for the blue emission). When lowering the dimension of the AlF_6_ octahedron, luminescence from KAlF_4_ (2D) and (NH_4_)_3_AlF_6_ (0D) is also discovered. Moreover, by changing B and X in the octahedral emissive unit of BX_6_, luminescence from AlCl_3_, Al(OH)_3_, Ga_2_O_3_, InCl_3_, and CdCl_2_ are also obtained. Similar to BX_6_ octahedron-based MHPs, the luminescence from AlF_3_ exhibit typical self-trapped exciton (STE) emission. Besides, the octahedron also brings close proximity of F atoms, resulting in weak through-space interaction of the *n* electrons in F atoms for clusterization-triggered emission (CTE, recently found in *n* electron-rich organics for excitation-dependent color-tunable phosphorescence^22–24^). It should be noted that it is normally the heavy atoms that drives the formation of triplet and phosphorescence in inorganics (e.g., previous Al-based luminescent materials, Supplementary Table 1). However, α-AlF_3_ contains only light elements. Therefore, the discovery here is interesting for both organic and inorganic phosphors. In addition, the intriguing color-tunable phosphorescence without extra sophisticated molecular design can be explored for facile UV light detection with visible colored afterglow emission as readout.

## Results

### Luminescence of α-AlF_3_

To investigate the luminescent properties of the BX_6_ octahedron, hexa-coordinated AlF_6_ was chosen first. Considering its outermost electronic structure (3*s*^2^3*p*^1^), aluminum is lightest atom to generate the octahedral structure. Meanwhile, F^‒^ is the smallest anion^25^ that can coordinate with Al^3+^. The corner shared octahedra of AlF_6_ results in the formation of three-dimensional network of α-AlF_3_ (Fig. 1). Normally, AlF_3_ is used as the electrolyte regulator in the aluminum smelting industry for increasing the melting point and conductivity. However, its photophysical properties are rarely studied.

**Fig. 2:**
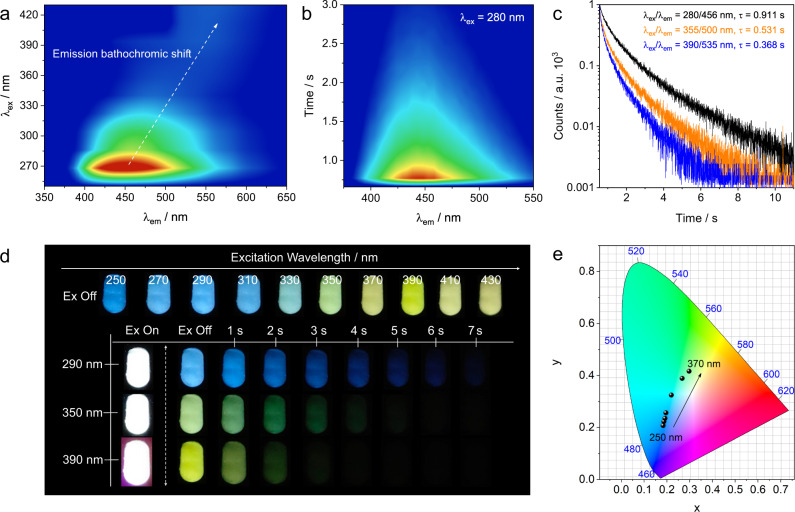
Luminescence properties of α-AlF_3_ (calcinated from AlF_3_·3H_2_O). **a** excitation- phosphorescence emission mapping of AlF_3_ (delay time: 40 ms); **b** time-resolved emission spectra (TRES, λ_ex_ = 280 nm) of AlF_3_; **c** lifetime decay profiles of phosphorescent emission excited at 280, 355, and 390 nm, respectively; **d** photographs taken under different excitation (250 to 430 nm) off and afterglow emission images excited at 290, 350 and 390 nm, respectively (the excitation-dependent afterglow can be observed in the excitation range from 250 to 510 nm at room temperature condition); **e** CIE coordinates of AlF_3_ phosphorescence under different excitation (250 to 370 nm).

Here, we found AlF_3_ exhibited exciting color-tunable luminescence at room temperature (Fig. 2a and Supplementary Fig. 3), irrespective of its origins (Supplementary Table 6 and Fig. 2). From the time-resolved emission spectra (TRES, Fig. 2b and Supplementary Fig. 11), the luminescence of α-AlF_3_ could be attributed to phosphorescence, with quantum yield (Φ_P_) of ~4.22% and lifetime up to ~0.9 s (λ_ex_ = 280 nm, Fig. 2c). The room-temperature afterglow of α-AlF_3_ could last more than 7 s (naked eye observable), and the excitation-dependent blue to yellow afterglow could be visualized clearly after ceasing the excitation (Fig. 2d, Supplementary Figs. 12–13, and Supplementary Movies 1–3). The color-tunable emission was also clearly revealed by the Commission Internationale de l’Eclairage (CIE) chromaticity coordinates (Fig. 2e). In addition, pure white light emission could be obtained (approaching CIE of 0.33, 0.33) when changing λ_ex_ from 270 nm to 390 nm (Fig. 2d and Supplementary Fig. 15).

To exclude the potential influence from trace impurities, direct synthesis of AlF_3_ through exposing aluminum metal of the highest purity available to HF vapor was carried out. As expected, similar emission properties were also obtained (Supplementary Figs. 6-7), confirming that the luminescence was exactly from AlF_3_. Furthermore, the purchased and as-prepared samples were processed by calcination, ball milling, and acid-washing, and no appreciable change of the luminescence property was received (Supplementary Figs. 8-10).

### Extending of the BX_6_ octahedral luminescent unit

**Fig. 3:**
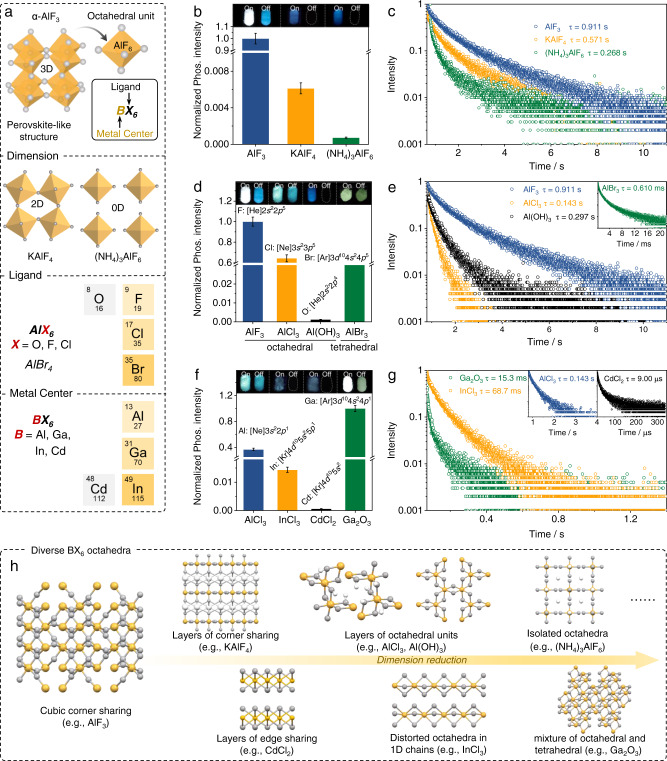
Investigation on the BX_6_ octahedral luminescent unit. **a**, schematic diagram of chemical structure of AlF_3_ and engineering of the octahedral basic unit; **b** and **c**, relative phosphorescence intensity and lifetime decay profiles (λ_ex_ = 280 nm) of the phosphorescence emission at 456, 480, 446 nm of AlF_3_, KAlF_4_, and (NH_4_)_3_AlF_6_, respectively; **d**, **e**, relative phosphorescence intensity and lifetime decay profiles (λ_ex_ = 280 nm) of the phosphorescence emission at 456, 486, 450, 496 nm of AlF_3_, AlCl_3_, Al(OH)_3_, and AlBr_3_, respectively; **f**, **g**, relative phosphorescence intensity, and lifetime decay profiles (λ_ex_ = 280 nm) of the phosphorescence emission at 486, 468, 460, 500 nm of AlCl_3_, InCl_3_, CdCl_2_, and Ga_2_O_3_, respectively; and **h**, summary of the diverse crystalline structures of the luminescent comprising BX_6_ octahedra. Error bars represent standard deviation (*n* = 3).

Since the 3D perovskite-like structure of α-AlF_3_ is constituted by the AlF_6_ octahedron, the basic unit of BX_6_ was further explored by adjusting the dimension of the octahedra (Fig. 3a). As shown in Figs. 3b and 3c, phosphorescence from KAlF_4_ (2D, layers of corner-sharing AlF_6_ octahedra and BCC coordinated K^+^) and (NH_4_)_3_AlF_6_ (0D, isolated AlF_6_ octahedra surrounded by NH_3_ ligands) was also collected (Supplementary Figs. 22 and 25), accompanied with similar excitation-dependent emission. The crystalline structure information (Supplementary Table 7) and XRD patterns confirmed that both KAlF_4_ and (NH_4_)_3_AlF_6_ were composed by the AlF_6_ octahedral unit and belonged to 2D and 0D metal halides structures (Supplementary Fig. 20), respectively. It should be noted that lowering the dimension of the octahedra resulted in largely decreased phosphorescence intensity and shortened lifetime (Fig. 3c), indicating that the linkage of the AlF_6_ octahedra also contributed to the observed phosphorescence.

Next, the ligand (X) of the BX_6_ basic unit was changed with other halogens, namely AlCl_3_ and AlBr_3_ (Fig. 3a). As shown in Fig. 3d and Supplementary Fig. 23, AlCl_3_ (cubic layered AlCl_6_ octahedra) still exhibited appreciable long-lived phosphorescence (Fig. 3e and Supplementary Fig. 26 and Supplementary Movie 4). Through altering halide composition from F to Br, their emission spectra are readily tunable from blue to yellow (Insets of Fig. 3d), which was similar with the emission tunable perovskite materials through halide engineering^26^. Although the outer space of Al is unable to accommodate six Br atoms to form stable hexadentate structure due to their relatively large atom radius discrepancy, AlBr_3_ (tetrahedral) is still luminescent, but with significantly reduced intensity and shortened lifetime (Fig. 3d, 3e). When altering the ligand from F to O, phosphorescence from the AlO_6_ (octahedral) derivatives (Al(OH)_3_ or Al_2_O_3_, 2D sheets of edge sharing Al(OH)_6_ octahedra) was also collected (Fig. 3d and Supplementary Fig. 23).

Upon changing the metal center of BX_6_, octahedral structure from Ga_2_O_3_ (mixture of GaO_6_ octahedral and GaO_4_ tetrahedral), InCl_3_ (distorted InCl_6_ octahedra in the 1D chains), and CdCl_2_ (2D sheets of edge-sharing CdCl_6_ octahedra) can be expected. Again, phosphorescence from these species was successfully collected (Fig. 3f and Fig. 3g, Supplementary Figs. 23 and 27, and Supplementary Movie 5 for Ga_2_O_3_ afterglow). It should be noted that there are diverse hexa-coordinate structures evolved from different bond angles (X-B-X), thus varied phosphorescence properties (excitation, emission, intensity, and lifetime, Fig. 3h)^27^.

### Luminescence mechanism of α-AlF_3_

**Fig. 4:**
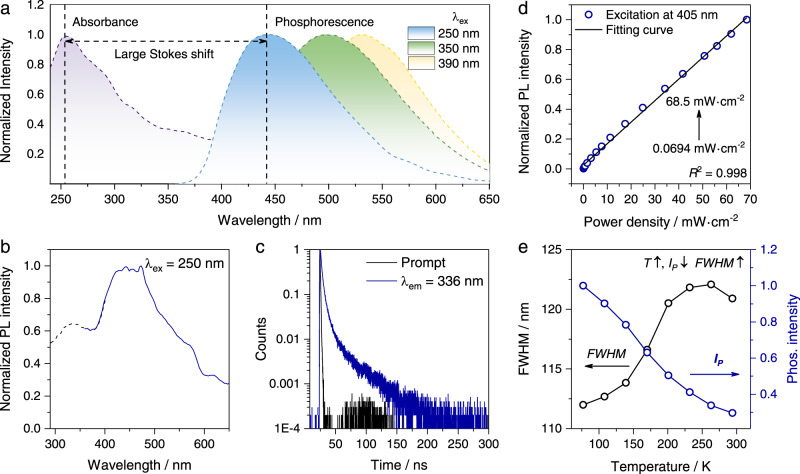
Investigation of the STE emission of AlF_3_. **a** Absorbance and phosphorescence spectra (λ_ex_ = 280, 350, and 390 nm, respectively.); **b** Normalized PL spectra (λ_ex_ = 250 nm, the dotted line was measured without long pass filters, while the dashed line with 341 nm long pass filter placed at the emission exit); **c** lifetime of AlF_3_ monitored at 336 nm (λ_ex_ = 280 nm); **d** plot of PL intensity as a function of excitation power density (λ_ex_ = 405 nm); **e** plots of FWHM and intensity of phosphorescence as a function of temperature (λ_ex_ = 280 nm; delay time: 40 ms).

The luminescence mechanism of the octahedral unit was investigated with the 3D network AlF_6_ (α-AlF_3_). Compared with MHPs, α-AlF_3_ showed the 3D perovskite-like structure, but without the insertion of alkali metal cations. These cations only contribute to lattice stabilization and does not participate in the formation of the frontier molecule orbitals^28^. The intrinsic emission of MHPs could be originated from free, bound and self-trapped excitons^29^. Among them, self-trapped exciton (STE) emission is a well-accepted mechanism to account for the broadband and large-stokes shift emission^30–33^. For α-AlF_3_, similar broad-band excitation-dependent blue to yellow emission was emerged, with full width at half-maximum (FWHM) of 120 nm and Stokes shift up to ~180 nm (Fig. 4a, emission spectra from λ_ex_ = 250 nm). In addition, a high energy narrow emission at 336 nm could be identified in the broad PL spectra (Fig. 4b), with lifetime of ~2.05 ns (Fig. 4c). According to the previous reports^34,35^, such emission could be ascribed to free excitons, which can be captured by the lattice distortion due to strong electron-phonon interaction in metal halides, resulting in the generation of STE.

The luminescence intensity of α-AlF_3_ exhibited a linear dependence on the excitation power density (more than three orders of magnitude, Fig. 4d), indicating that the emission is originated from photogenerated exciton (self-trapped) rather than permanent defect, the latter of which would show saturated PL intensity upon increasing the excitation power density^36^. Also, the emission band was not changed upon altering the excitation power density (Supplementary Fig. 16), further excluding the possibility of other emissive defects. Moreover, temperature-dependent phosphorescence spectra and cryogenic lifetime were collected (Supplementary Figs. 17, 18). The emission intensity was enhanced upon lowering the temperature (294 K→77 K), accompanied by a decrease in the FWHM (Fig. 4e), which was consistent with the characteristics of electron-lattice coupling^37^. Therefore, all these above spectral features agreed well with STE.

**Fig. 5:**
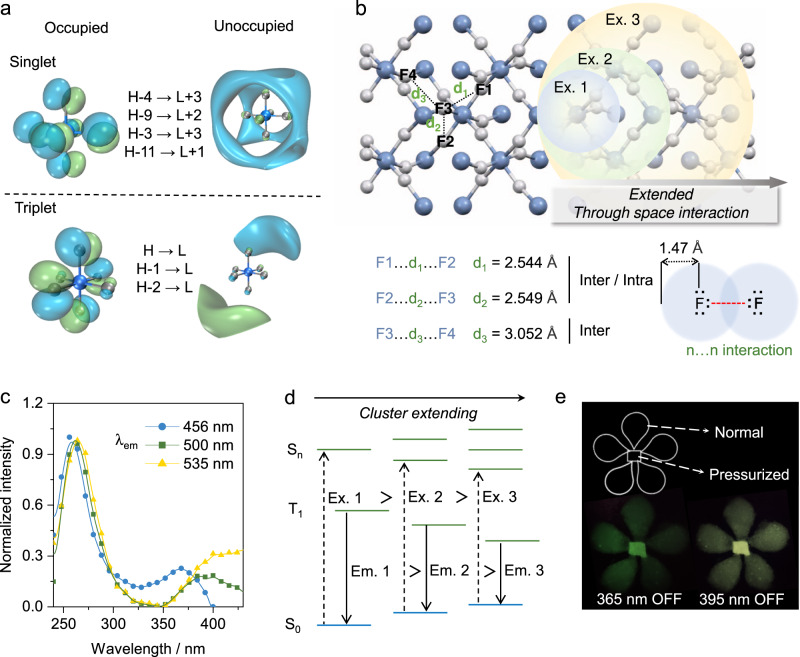
Investigation of the phosphorescence of AlF_3_. **a** the TD-DFT calculated isosurfaces of occupied and unoccupied orbitals of excited singlet state with maximum oscillator strength and lowest triplet excited state in AlF_6_ octahedral unit. **b** structural analysis of α-AlF_3_ (crystalline structure from ISCD 68826). **c** excitation spectra (λ_em_ = 456, 500, and 535 nm, respectively.) of α-AlF_3_ **d** schematic energy level diagram of AlF_3_ from one octahedron to network. **e** Photograph of AlF_3_ afterglow with a flower pattern, consisting of untreated and pressure-treated α-AlF_3_, respectively.

On the other hand, although α-AlF_3_ owns distorted 3D perovskite-like structure, its room-temperature phosphorescence exhibited interesting excitation-dependent feature. To further illustrate the mechanism, the transitions of AlF_3_ was investigated through theoretical calculations. Considering that the BX_6_ octahedra can be luminescent in isolated, corner-shared, and distorted structures, a single unit of AlF_6_ was calculated with the time-dependent density functional theory (TD-DFT)^38,39^. The calculated excitation energy with the highest oscillator strength and the emission energy from the lowest triplet state (T_1_) to the ground state (S_0_) are 4.76 eV and 3.36 eV, respectively, indicating potential large Stokes shift. Next, the natural transition orbitals (NTO) were analyzed with Multiwfn^40^. As shown in Fig. 5a and Supplementary Fig. 29, the transition with highest oscillator strength happened from the un-bonding *n* electron of F to the antibonding orbitals composed by the *s* orbital of Al and the *p* orbital of F. Meanwhile, such transition exhibited a typical *n*→σ* character, which is consistent with the deep UV absorption of AlF_3_. For phosphorescence transition (T_1_→S_0_), the frontier orbitals comprise F 2*p* (HOMO) as well as Al 3*p* and F 2*p* (LUMO). According to the selection rule for electronic spectra^41^, the electron transition of *p*-*p* (similar to *f*-*f* transition of lanthanides) orbitals is parity-forbidden, which is essential for the long-lived phosphorescence of AlF_3_. For the other BX_6_ octahedra, similar transitions (*n*→σ*) could also be identified and their T_1_→S_0_ transitions agreed well with experimental results (Supplementary Figs. 32–36).

Recently, a number of excitation-dependent color-tunable phosphors featuring *n*→σ* transitions were reported, in which weak through-space interaction (TSI) was identified from the rich *n* electrons of the heteroatoms in the phosphors (cluster-induced emission, CIE)^22–24^. Structurally, α-AlF_3_ is a nonconjugated system without through-bond conjugation, but F atoms with n electrons are abundant to form corner sharing networks (Fig. 5b). Importantly, the distances between the adjacent F atoms in and between the AlF_6_ octahedra (e.g., intra/inter: ~2.544 and ~2.549 Å; inter: ~3.052 Å) generally fall in the van der Waals radii of F atom (1.47 Å)^42^. Therefore, there is possible van der Waals F···F interaction, thus leading to effective electron cloud overlap of the n electrons for *n*→σ* transition^23^. It should be noted that F atoms also exist in NaF and KF, but no such phosphorescence could be found (Supplementary Fig. 24), further highlighting the importance of BX_6_ octahedron.

Spectrally, the excitation spectra of α-AlF_3_ contains two peaks, the short wavelength of which matches with its absorption, while those at longer wavelengths red shifted upon increasing the emission wavelengths (Fig. 5c). Besides, the excitation peaks at wavelength longer than the absorption were generally not detectable as a significant feature in the absorption spectra (Fig. 3a), which is also a typical sign of CTE. Moreover, as further calculated with the quantum mechanics and molecular mechanics (QM/MM) method, the energy gap decreased as increasing the number of AlF_6_ octahedra (increasing the sizes of the clusters, Supplementary Fig. 30). Therefore, different clustering states may result in varied energy gaps (Fig. 5d), thereby excitation-dependent and color-tunable phosphorescence.

The weak interaction between AlF_6_ octahedra in α-AlF_3_ was further investigated with Atoms in Molecules (AIM) analysis^43^. As shown in Supplementary Fig. 31, in addition to traditional bond path in the octahedron (Al-F), there is also plenty of through-space interaction path (F···F interaction) as the AlF_6_ octahedra system extended. Experimentally, upon high pressure treatment (900 MPa) to strengthen the weak intermolecular Van-der Waals interaction^44^, the emission brightness of α-AlF_3_ was increased somewhat (Fig. 5e, Φ_P_ increase from ~4.22% to ~5.46%), while the multicolor emission profiles of α-AlF_3_ were not disturbed (Supplementary Fig. 19). Therefore, such interactions are expected to facilitate electron communications between the *n* electrons of F atoms and rigidify the system for efficient phosphorescence.

On the basis of the above analysis, similar excitation-dependent but decreased phosphorescence intensity from KAlF_4_ and (NH_4_)_3_AlF_6_ (and also other n electrons rich compounds featuring BX_6_ octahedra, Fig. 3h) can thus be expected. Lowering the dimension of the AlF_6_ octahedra from 3D corner-sharing (α-AlF_3_) to layered (KAlF_4_) and isolated ((NH_4_)_3_AlF_6_) would decrease the inter-octahedra F···F interaction, thus weakening the through-space interaction (Supplementary Fig. 21). Moreover, the 3D corner-sharing structure may also rigidify the AlF_6_ octahedra, which is beneficial for stabilization of the excited triplet states. Therefore, compared with α-AlF_3_, lowering the dimension of the AlF_6_ octahedra would result in sharply decreased phosphorescence intensity and lifetime in KAlF_4_ and (NH_4_)_3_AlF_6_. For other BX_6_ octahedron-containing materials, different phosphorescence performances would also be expected, due to their differences in B, X, and connection of the octahedra (Fig. 3h).

### RTP of α-AlF_3_ for UV wavelength detection

**Fig. 6:**
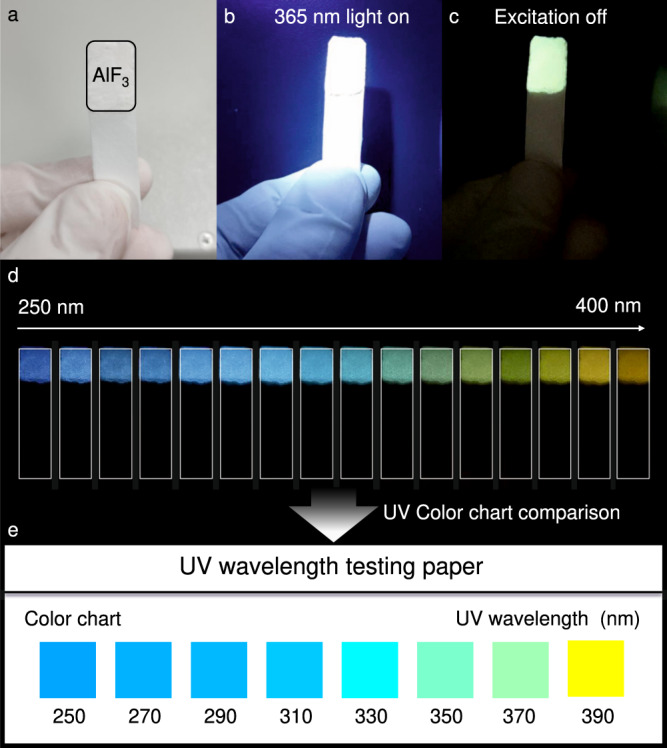
UV wavelength detection with AlF_3_-loaded testing paper. **a** illustration of the testing paper coated with AlF_3_; **b** a beam of UV excitation irradiated on the testing paper; **c** AlF_3_-testing paper phosphorescence demonstration after ceasing 365 nm excitation; **d** multicolor phosphorescence from the AlF_3_-tesing paper after ceasing a series of UV excitation; **e** color chart of UV wavelength testing paper.

Considering that the long-lived phosphorescence from AlF_3_ is color-tunable in the visible range and excitation-dependent (particularly in the UV range), it was simply and conveniently explored for ultraviolet wavelength detection in a testing paper manner. As demonstrated in Fig. 6a, after mixed with Aloe vera gel for fixing, AlF_3_ was coated on the filter paper. Upon UV irradiation, white luminescence from AlF_3_ was excited (Fig. 6b). After ceasing of UV excitation, visible afterglow images could be obtained (Fig. 6c and Supplementary Movie 6). Notably, upon excited with UV light of different wavelengths, the afterglow emission varied from blue to orange, which could be further compared with standard color chart to confirm UV excitation wavelength (Fig. 6d and Fig. 6e). Such application provided a method for unknown UV wavelength detection and offered rapid and simple standard screening and testing of commercially UVA and UVB ultraviolet lamps available.

## Discussion

**Fig. 7:**
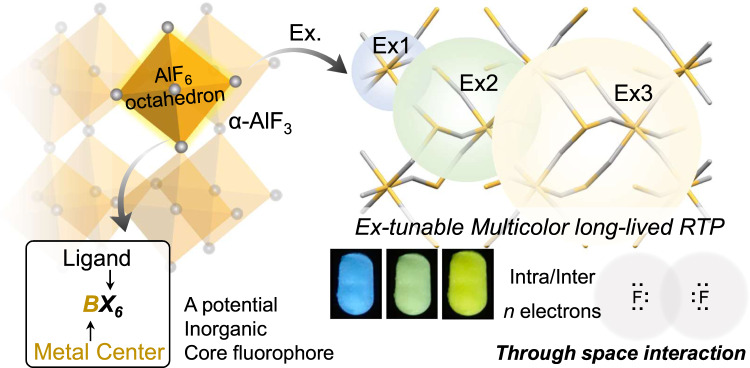
Summary on the BX_6_ octahedra-based luminescence. Here, the luminescence mechanism of perovskite-like α-AlF_3_ (and also others) was ascribed to clusterization-triggered emission of *n* electron-rich AlF_6_ octahedrons, thus exhibiting excitation-tunable multicolor long-lived RTP.

In this work, perovskite-like octahedral BX_6_ was proposed a basic unit for luminescent inorganic materials. In this regard, we found that α-AlF_3_ (constituted by vertex sharing AlF_6_ octahedral unit) exhibited long-lived color-tunable phosphorescence emission, which could last up to 7 s and be observed by naked eyes. Besides, by lowering the dimension of the AlF_6_ octahedron and changing B and X in the BX_6_ octahedron, luminescence from KAlF_4_, (NH_4_)_3_AlF_6_, AlCl_3_, Al(OH)_3_, Ga_2_O_3_, InCl_3_, and CdCl_2_ were also obtained. The phosphorescence of AlF_3_ could also be explained with the well-accepted STE mechanism of perovskite BX_6_ octahedra, together with the clusterization-triggered emission of *n* electron-rich phosphors. Therefore, BX_6_ octahedron may be a universal structure motif for inorganic luminescent materials (Fig. 7).

For a long time, it has been well-accepted that there are core structures for organic luminescent materials, and their general photophysical properties can thus be interpreted. For inorganic luminescent materials, such core structure remains elusive. Our results here indicated that the boundary between the above two may be somewhat vague. Inorganic AlF_3_ (also AlCl_3_, Ga_2_O_3_, and etc.), featured with perovskite-like octahedral BX_6_ basic unit, can emit long-lived phosphorescence very much similar to CTE from non-conjugated organic luminophores^22^. Moreover, typical *n*→σ* transition was found in the single unit of AlF_6_, which is also the typical character of CTE. Therefore, future development of luminescent materials integrating of both organic and inorganic luminescence mechanisms is expected to be appealing, particularly in MOFs containing both inorganic and organic units.

Last, in the long history of the afterglow phosphor family, the IIIA group elements contributed heavily (Supplementary Table 1, 2), particularly Al- and Ga-containing materials (e.g., the well-known SrAl_2_O_4_:Eu^2+^-Dy^3+13^ and ZnGa_2_O_4_:Cr^3+^ ^45^). However, the luminescence center are mostly lanthanides or transition metal ions^46^ (e.g., Mn^2+^, and Cr^3+^). Here, Al- and Ga-containing afterglow phosphors with ~4% QY (e.g., AlF_3_ and Ga_2_O_3_) are discovered. Particularly, the luminescence is come from the Al- and Ga-involved octahedral unit, not the dopants. Therefore, there is still much room for the exciting IIIA group chemistry in luminescent materials design.

## Methods

### Preparation of AlF_3_

AlF_3_ was prepared through calcination of AlF_3_·3H_2_O (99.9%, Macklin) at 350 °C for 3 h. To eliminate potential organic impurities, other AlF_3_ samples were also subjected to the same calcination process as above. In addition, direct synthesis of AlF_3_ was carried out through exposing aluminum metal (Aladdin, 99.999%, the highest purity available) to HF vapor from hydrofluoric acid (Macklin, 49wt. % in H_2_O, 99.99998%) for 3 h, followed by calcination at 350 °C for 3 h.

### Phosphorescence measurements

Phosphorescence spectra were collected on HORIBA FluoroMax-4P with a delay time of 40 ms. For the temperature-dependent emission spectra, a model Optistat CF2 liquid nitrogen chamber (Oxford Instruments) was used and coupled with the FluoroMax-4P spectrofluorometer. Phosphorescence lifetime and the time-resolved emission spectra (TRES) were collected on HORIBA FluoroLog-3 spectrofluorometer with 280, 355, and 390 nm spectraLED as the excitation sources, respectively. The absorption spectra of solid sample were measured on Shimadzu UV-3600 with an integrating sphere unit. The phosphorescence quantum yield (Ф_*p*_) of samples were measured in an integrating sphere (IS80, Labsphere) using 2-fluorophenylboronic acid (Ф_*p*_ = 0.98%^47^) as the reference 48.

### Afterglow images

The samples were excited with light selected from the Xe lamp in the Fluolog-3 spectrofluorometer (250 to 510 nm)^48^. The shutting of the excitation was controlled by instrumental software. The camera started to record video for 10 s after the excitation was turned off immediately.

### Theoretical calculation

Theoretical calculations for the octahedral unit were performed on Orca program package (Revision 4.1.1). The ground states (S_0_) were fully optimized by M062X with ma-def2-TZVP basis set. The excitation energies in the n-th singlet (S_n_) and n-th triplet (T_n_) states were obtained using the time-dependent density functional theory (TD-DFT) method based on an optimized molecular structure. In order to explore detailed excited state properties, NTO (Natural transition orbitals) analysis and hole-electron analysis method were further employed using Multiwfn program. For different cluster sizes of the AlF_6_ octahedra, QM/MM method was applied to obtain optimized structure of isolated, layered and cubic corner sharing AlF_6_ octahedra performed in the Gaussian 09 package^49^. The central AlF_6_ octahedra were treated at the (TD) M062X/6-31G+ (d,p) level, while the surrounding molecules were treated with universal force field (UFF).

For the weak interaction between AlF_6_ octahedra, AIM analysis was carried out, which dictates the form of atoms in molecules and analyzes the topology of electron density. Typically, the results could be characterized by “critical points” of, namely bond (BCP), ring (RCP) and cage (CCP) critical points, representing the extreme points of electron density on the bond paths, centers of rings, and enclosed space formed by rings, respectively. It was further employed using Multiwfn program. The molecular orbitals (MO) and AIM models were all displayed using VMD.

### UV wavelength testing paper

α-AlF_3_ was mixed with Aloe vera gel with mass ratio of ~1: 1, followed by fixing on the filter paper to make the mixture evenly distributed and drying. For UV wavelength detection, the testing paper was first subjected to UV excitation for 5 seconds, then the afterglow images were either naked eye observed or photo taken with a camera.

## Supplementary information

Supplementary Information

Peer Review File

Description of Additional Supplementary Files

Supplementary Movie 1

Supplementary Movie 2

Supplementary Movie 3

Supplementary Movie 4

Supplementary Movie 5

Supplementary Movie 6

## Data Availability

The data supporting the findings of this study are available within the paper and the Supplementary Information. Source data are provided with this paper.

## Source data

Source Data
