# Supplementary material for: Multicolor ultralong phosphorescence from perovskite-like octahedral α-AlF3
Source: Nat Commun. 2022 Sep 29;13:5712. doi: 10.1038/s41467-022-33540-1 (PMC9522726; doi:10.1038/s41467-022-33540-1)
Supplement: Supplementary file 1 — Supplementary Information [file 41467_2022_33540_MOESM1_ESM.pdf]

Supplementary Information

Multicolor Ultralong Phosphorescence from Perovskite-Like Octahedral α-AlF_3

Peisheng Cao,[1] Haoyue Zheng,[2] Peng Wu[1, 2,*]

[1] College of Chemistry, and [2] Analytical & Testing Center, Sichuan University, Chengdu, 610064, China

*Corresponding Authors' E-mail: wupeng@scu.edu.cn

Table of contents

Section S1. Research Background 3

Section S2. Experimental Section 5

S2.1 Materials 5

S2.2 Apparatus 6

S2.3 Lifetime Fitting 7

Section S3. Aluminum Fluoride 8

S3.1 Confirmation of the RTP from α-AlF_3 8

S3.2 Afterglow of α-AlF_3 18

S3.3 Additional photophysical data of α-AlF_3 23

Section S4. The Octahedral unit derivatives 26

S4.1 Structures 26

S4.2 Photophysical properties 29

S4.3 Afterglow images 34

Section S5. Theoretical calculation 36

S5.1 Theoretical calculation of AlF_3 36

S5.2 Theoretical calculation of octahedral unit derivatives 39

Supplementary References 44

Section S1. Research Background

Supplementary Table 1. Summary of the aluminum-containing luminescent materials.

Luminescent species	Luminescent origin	Role of aluminum	Reference
$SrAl_2O_4$: Eu^{2+}, Dy^{3+}	Lanthanide ions	Inorganic host	[1]
$CaAlO_4$: Ce^{3+}, Mn^{2+}	transition metal ions	Inorganic host	[2]
$Y_3Al_5O_{12}$: Eu, Si	Charge transfer from Eu^{2+} to Si^{4+}	Inorganic host	[3]
$MgAl_2O_4$	V_K^{3+} center	Formation of defect	[4]
$CuAlSe_2$	Intrinsic vacancies	Formation of aluminum vacancies	[5]
AlN: Mn^{2+}	Mn^{2+}	Inorganic host	[6]
AlN	Intrinsic vacancies	Formation of aluminum vacancies	[7]
Al_2O_3	Defect	Formation of defect	[8]
Anodic aluminum oxide films (AAOF): Eu^{3+}, Tb^{3+}	Lanthanide ions	Inorganic matrix	[9]
Carbon dots (CDs) in aluminum sulfate	Carbon dots	Inorganic matrix	[10]
Cu_8 NC-Al^{3+}	Cu_8 NC	Aluminum ion triggered aggregation induced emission	[11]
HPB-6CO_2Na-Al^{3+}	Organic molecule	Aluminum coordination triggered aggregation induced emission	[12]
Alq_3	Organic ligand	Aluminum coordination triggered emission	[13]
Aluminum-based MOF (eg: CAU-10-CHO, CAU-10-V-H)	Intrinsic luminescence	Formation of luminescent center	[14,15]

Supplementary Table 2. Summary of the gallium-containing luminescent materials.

Luminescent species	Luminescent origin	Role of aluminum	Reference
$LiGa_5O_8$: Cr^{3+}	Cr^{3+}	Inorganic host	[16]
$M_3Ga_2Ge_4O_{14}$: Cr^{3+} (M = Sr or Ca)	Cr^{3+}	Inorganic host	[17]
$Zn_3Ga_2Ge_2O_{10}$: Cr^{3+}	Cr^{3+}	Inorganic host	[18]
$KGaGeO_4$: Bi^{3+}	Bi^{3+}	1. Inorganic host<br>2. Formation of vacancy	[19]
$ZnGa_2O_4$: Mn^{2+} (Cr^{3+})	Doped ions	Inorganic host	[20]
$ZnGa_2O_4$	Self-activated center of the octahedral Ga-O group	Formation of center	[21]
GaN: Eu	Eu^{3+}	Inorganic host	[22]
Ga_2O_3: Mn (Cr, Co, Sn, rare-earth ions)	doped ions	Inorganic host	[23]
β-Ga_2O_3	①Self-trapped exciton<br>②vacancy	Formation of exciton/vacancy	[24-26]
γ-Ga_2O_3 Nanocrystals	donor-acceptor pair	Formation of acceptor	[27,28]
$(HDADD)_2(BPDC)_{0.5}$-$[Ga_3(OH)_2(HPO_4)_4]$ DADD = 1,12-diaminododecane, BPDC = 4,4′-biphenyldicarboxylate	organic templates	Inorganic matrix	[29]

Section S2. Experimental Section

S2.1 Materials

The detailed information of the materials was given in Supplementary Table 1.

Supplementary Table 3. The information of the sample used in this work.

Name	CAS No.	Specification	Supplier
AlF_3	7784-18-1	99.99%	Aladdin
		99.9%	Macklin
		99.9%	Qurchem
		99%	Aladdin
$AlF_3 \cdot 3H_2O$	15098-87-0	99.9%	Macklin
		CP	SCR
$KAlF_4$	14484-69-6	CP	Aladdin
$(NH_4)_3AlF_6$	7784-19-2	98%	Aladdin
Na_3AlF_6	13775-53-6	99.99%	Aladdin
$Al(OH)_3$	21645-51-2	99.99%, 2～10 μm	Aladdin
Al_2O_3	1344-28-1	99.99%, γ phase, 20 nm	Aladdin
$AlCl_3$	7446-70-0	99.99%	Macklin
$AlBr_3$	7727-15-3	99.999%	Aldrich
$CdCl_2$	10108-64-2	99%	Aladdin
Ga_2O_3	12024-21-4	99.99%	Aladdin
$InCl_3$	10025-82-8	99.99%	Aladdin
Al	7429-90-5	99.999%	Aladdin
Hydrofluoric acid	7664-39-3	49wt. % in H_2O 99.99998%	Macklin

S2.2 Apparatus

Supplementary Table 4. The instrumental information used in this work.

Characterization items	Type	Manufacturer
phosphorescence spectrum	Fluoromax-4 spectrofluorometer	Horiba Jobin Yvon, USA
Photoluminescence spectrum<br>Phosphorescence lifetime & QY	Fluolog-3 spectrofluorometer	Horiba Jobin Yvon, USA
Chemical Luminescence spectrum	MPI-E electrochemiluminescence analyzer detector<br>excitation: LED: 365 nm	Remex, China
Chemiluminescence Imaging	Azure C300<br>excitation: LED: 365 nm	Azure, USA
UV/Vis spectrum	UV-3600	Shimadzu, Japan

S2.3 Lifetime Fitting

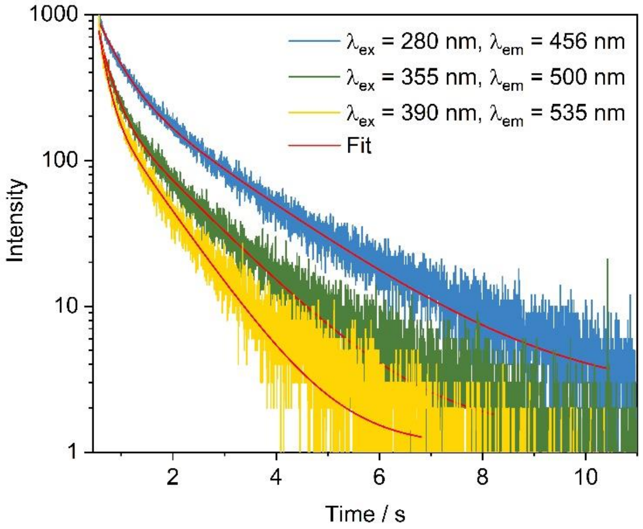

Supplementary Figure 1. Lifetime decay profiles of AlF_3 phosphorescent emission excited at 280, 355, and 390 nm and their corresponding fitted curves.

All lifetime measurements are fitted by using 2 exponentials, The decay as a function of time is given by:

$$F(t) = A + B_1 \exp\left(-t/_{T_1}\right) + B_2 \exp\left(-t/_{T_2}\right)$$

Supplementary Table 5. Fitted parameters of AlF_3 lifetime measurement.

Fitted parameters	λ_{ex} = 280 nm<br>λ_{em} = 456 nm	λ_{ex} = 355 nm<br>λ_{em} = 500 nm	λ_{ex} = 390 nm<br>λ_{em} = 535 nm
T1 / s	0.4081773	0.2287004	0.1546947
T2 / s	1.770778	1.230099	0.8639073
A	2.486552	1.368256	1.105836
B1	0.63	0.70	0.70
B2	0.37	0.30	0.30
Average Life Time / s	0.911	0.531	0.368
CHISQ	1.166688	1.252574	1.15616

Section S3. Aluminum Fluoride

S3.1 Confirmation of the RTP from α-AlF_3

Supplementary Table 6. Summary of the emission properties of AlF_3 from different manufactories.

Manufacture	Purity	λ_{ex} = 280 nm		λ_{ex} = 355 nm	
		$\lambda_{em, P}$ / nm	τ_P / s	$\lambda_{em, P}$ / nm	τ_P / s
Aladdin	99.99%	456	0.803	500	0.458
	99%	462	0.929	534	0.412
Qurchem	99.9%	454	0.858	526	0.470
Macklin	99.9%	458	0.937	538	0.370

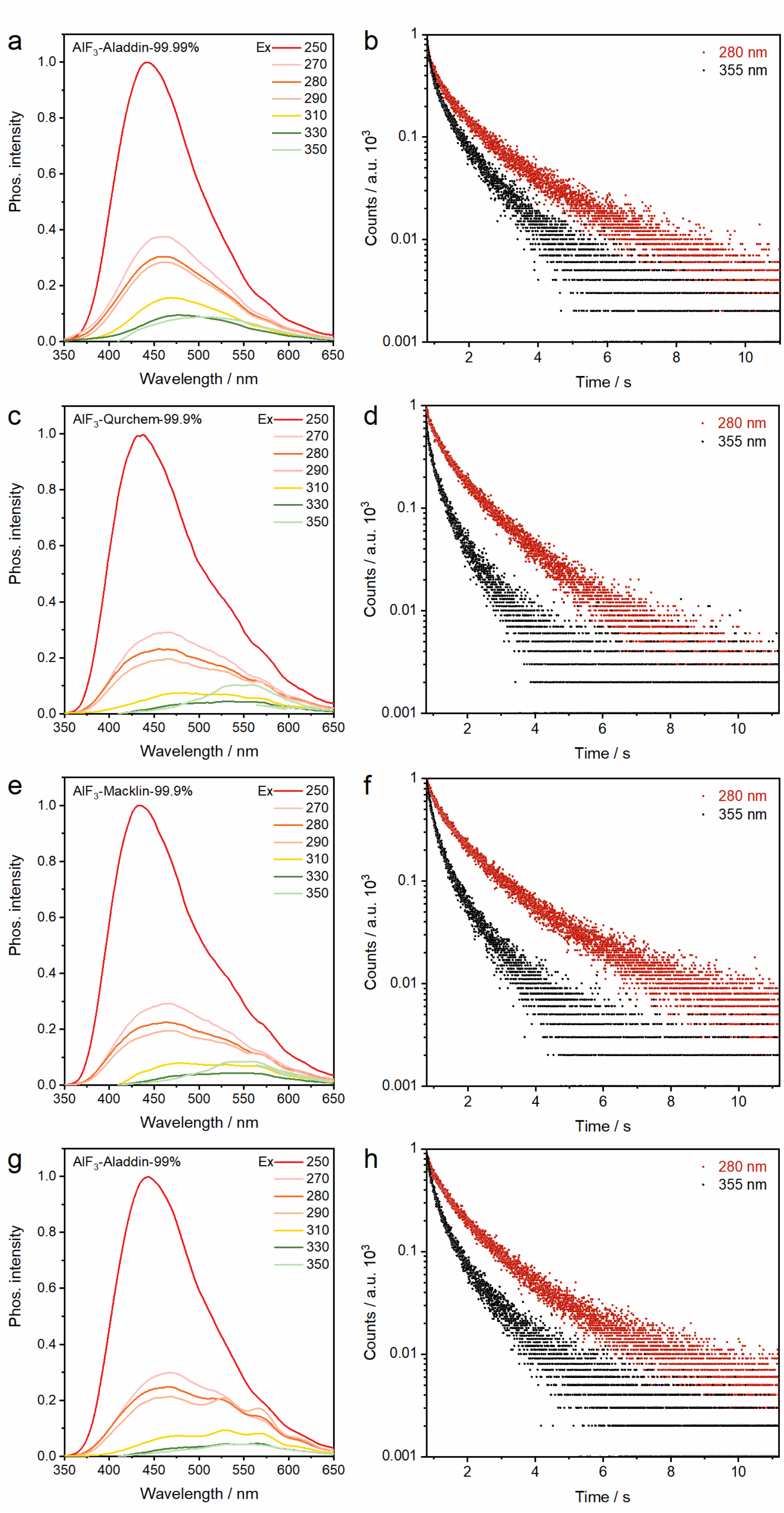

Supplementary Figure 2. The excitation-dependent phosphorescence spectra **(a, c, e, g)** with delay time of 40 ms and phosphorescent lifetime **(b, d, f, h)** of AlF_3 from different manufactories.

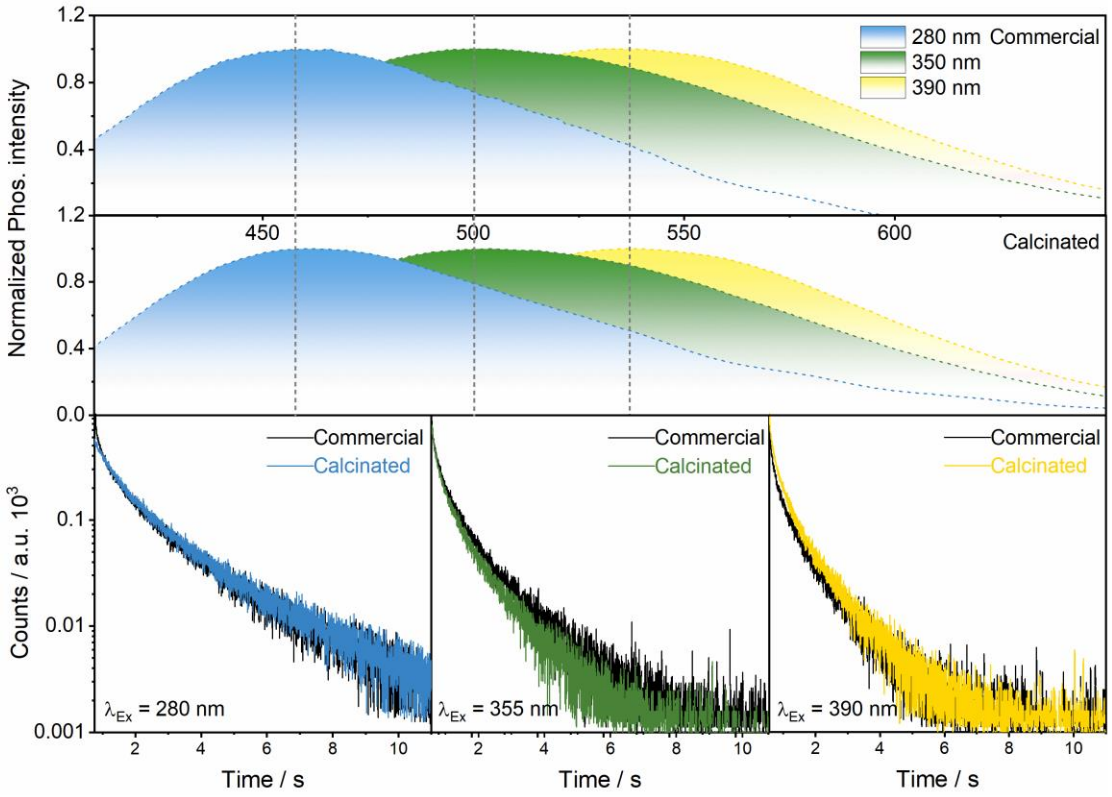

Supplementary Figure 3. Normalized phosphorescence spectra (delay time: 40 ms) and phosphorescent lifetime of commercial AlF_3 and synthetic sample featuring single R3c lattice phase (α-AlF_3) from $AlF_3{\cdot}3H_2O$ calcination excited by 280, 350 and 390 nm, respectively.

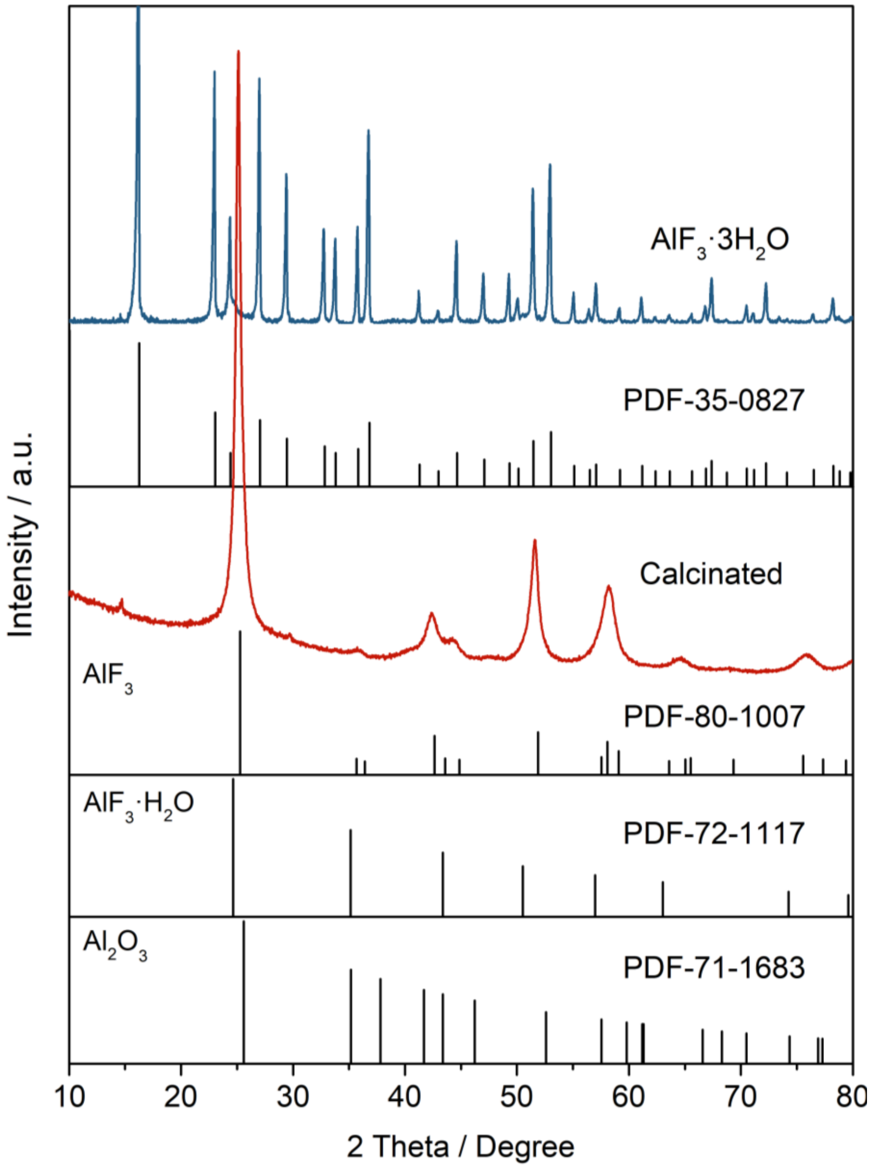

Supplementary Figure 4. XRD patterns of original $AlF_3 \cdot 3H_2O$ and the sample processed by calcination.

The purchased sample owned β-$AlF_3 \cdot 3H_2O$ crystalline phase. After calcination, the result showed that there only existed single R3c lattice phase (α-AlF_3) and did not comprise distinct lattice phase and impurities such as Al_2O_3 or AlF_3 hydrates.

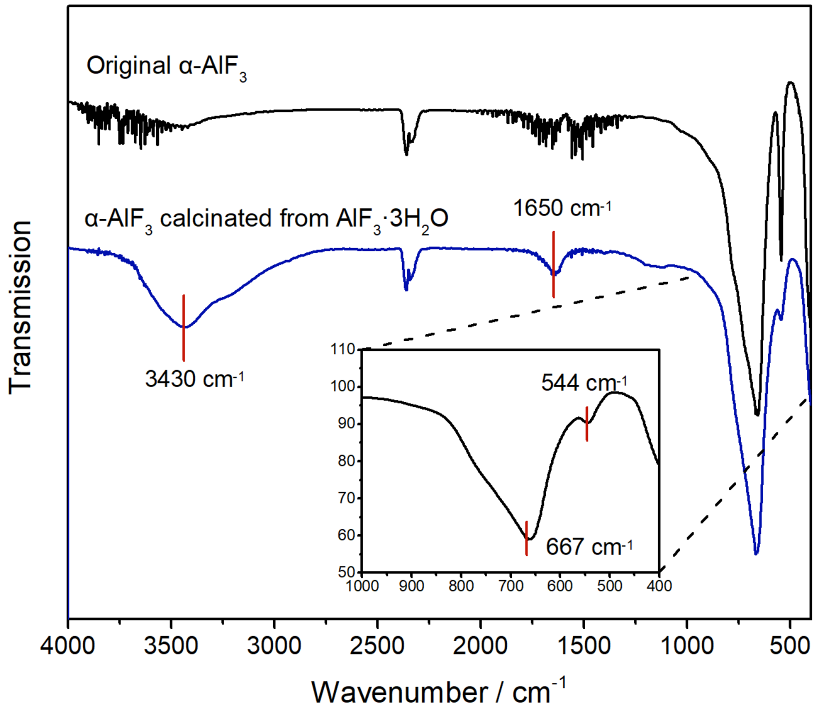

Supplementary Figure 5. IR spectra of original AlF_3 and the calcinated sample from $AlF_3·3H_2O$.

The IR spectra showed α-AlF_3 667 cm^{-1} stretching vibrational band with a characteristic 544 cm^{-1} weak band, demonstrating that there only existed vertex sharing AlF_6 lattice structure.[30] However, additional large envelop detected in the 2800-3700 cm^{-1} range was also observed, which could be attributed to ν_{O-H} and deformation mode of water molecule δ_{H2O} located at 1650 cm^{-1}. All the above phenomena confirmed the occurrence of hydrogen bonding inside the structure. Indicating that the enhanced luminescence of α-AlF_3 could be ascribed to the decrease of nonradiative relaxation based on hydrate stabilization, and similar behavior also occurs for some other metal halides cluster,[31] molecular cocrystal,[32] and carbon dot[33] systems.

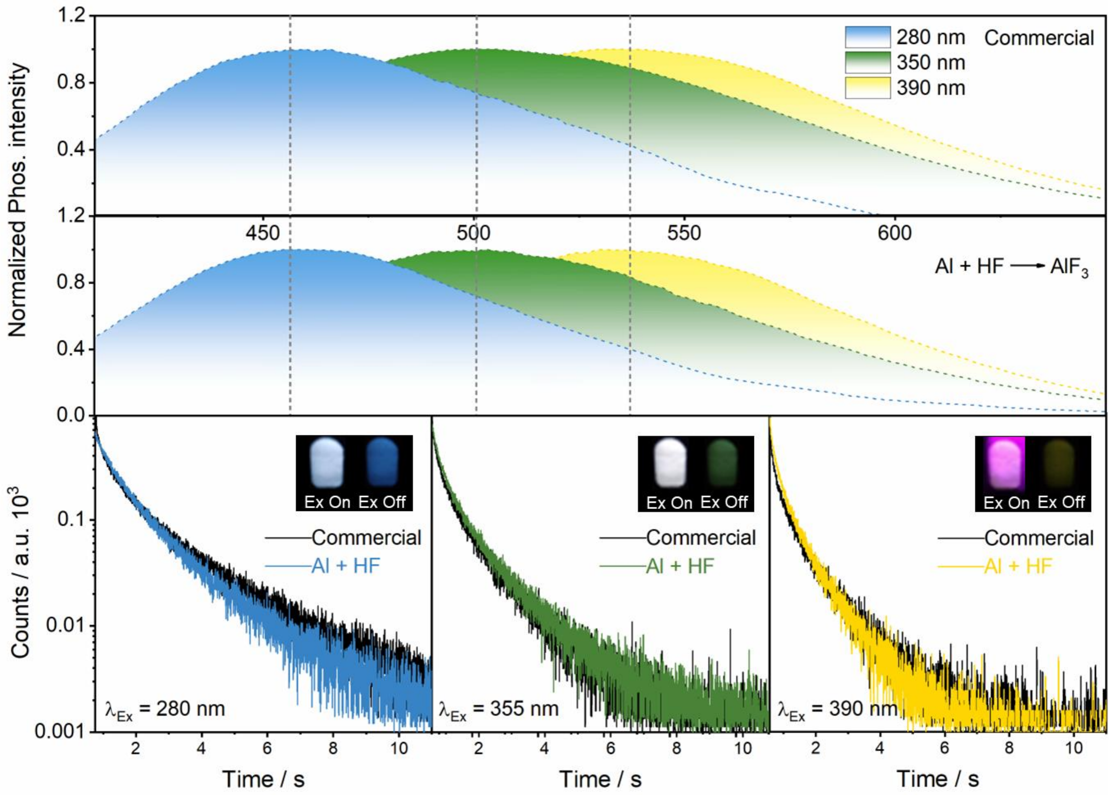

Supplementary Figure 6. Normalized phosphorescence spectra and phosphorescent lifetime of original AlF_3 and prepared samples from treating aluminum metal (Aladdin, 99.999%) with hydrofluoric acid (Macklin, 49wt. % in H_2O, 99.99998%) excited by 280, 350 and 390 nm, respectively.

These emission profiles and lifetimes for calcinated sample were invariable compared with AlF_3 original sample to confirm these phenomena were exactly originated from intrinsic emission.

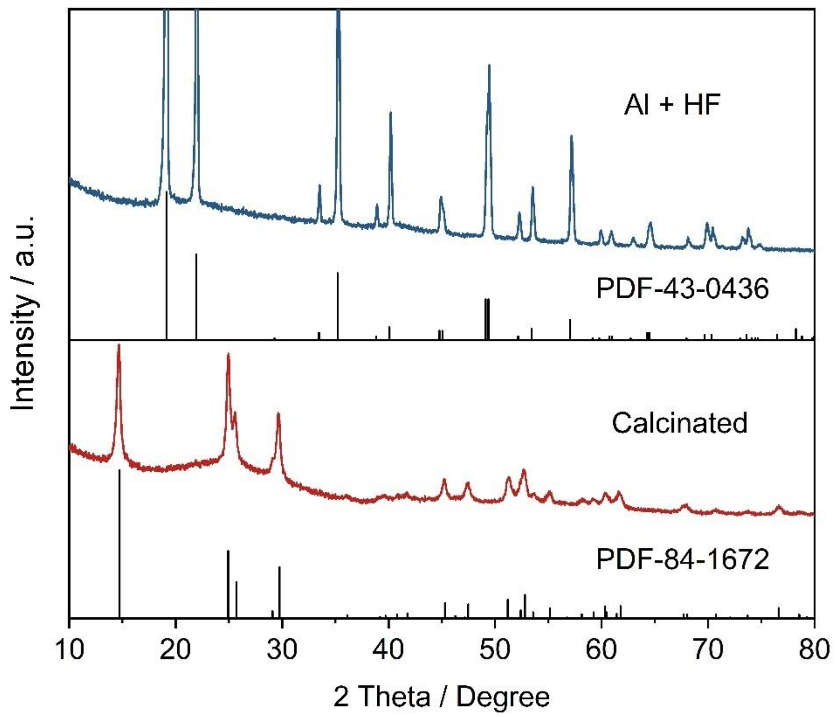

Supplementary Figure 7. XRD patterns of sample prepared by treating aluminum metal with hydrofluoric acid and processed by further calcination.

The result showed that the synthetic product from aluminum metal and hydrofluoric acid owned α-$AlF_3·3H_2O$ crystalline phase. After calcination, the result showed that there only existed β-AlF_3 lattice phase and further demonstrated the existence of AlF_6 unit as BX_6 octahedron could be deemed as luminescent core structure, irrespective of crystalline phase.

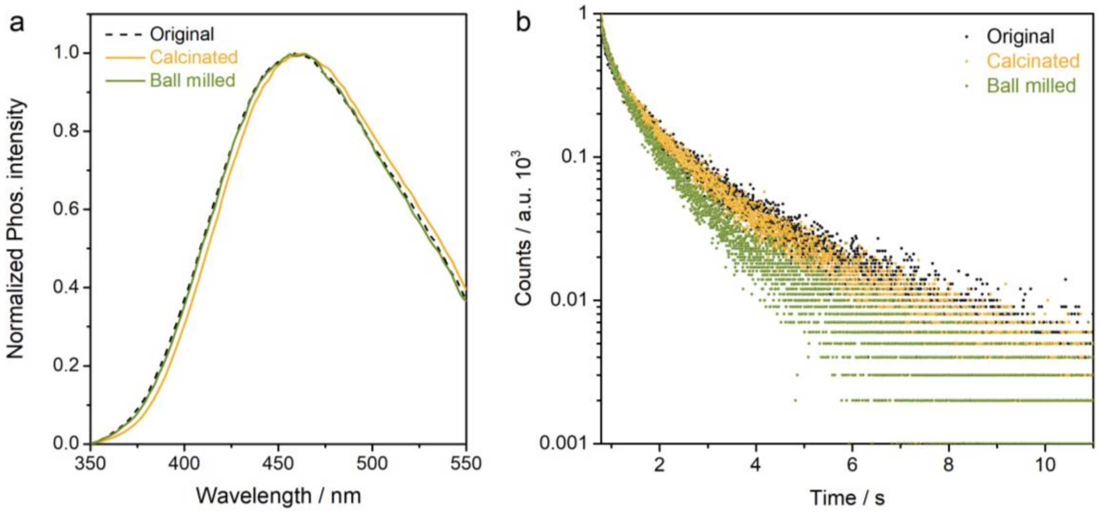

Supplementary Figure 8. The phosphorescence spectra **(a)** with delay time of 40 ms and phosphorescence lifetime **(b)** excited by 280 nm of AlF_3 before and after calcination/ball milling.

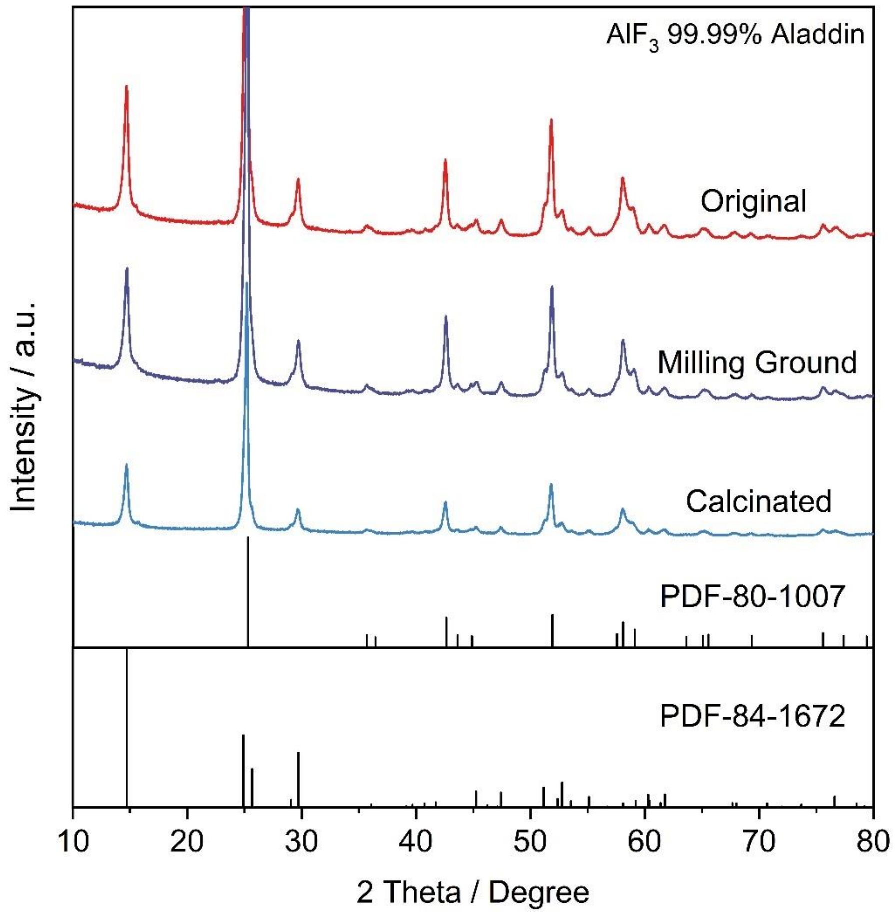

Supplementary Figure 9. XRD patterns of the original AlF_3 and samples processed by ball milling and calcination, respectively.

The PDF files correspond to AlF_3 with space group of R3c (PDF-80-1007) and Cmcm (PDF-84-1672), respectively.

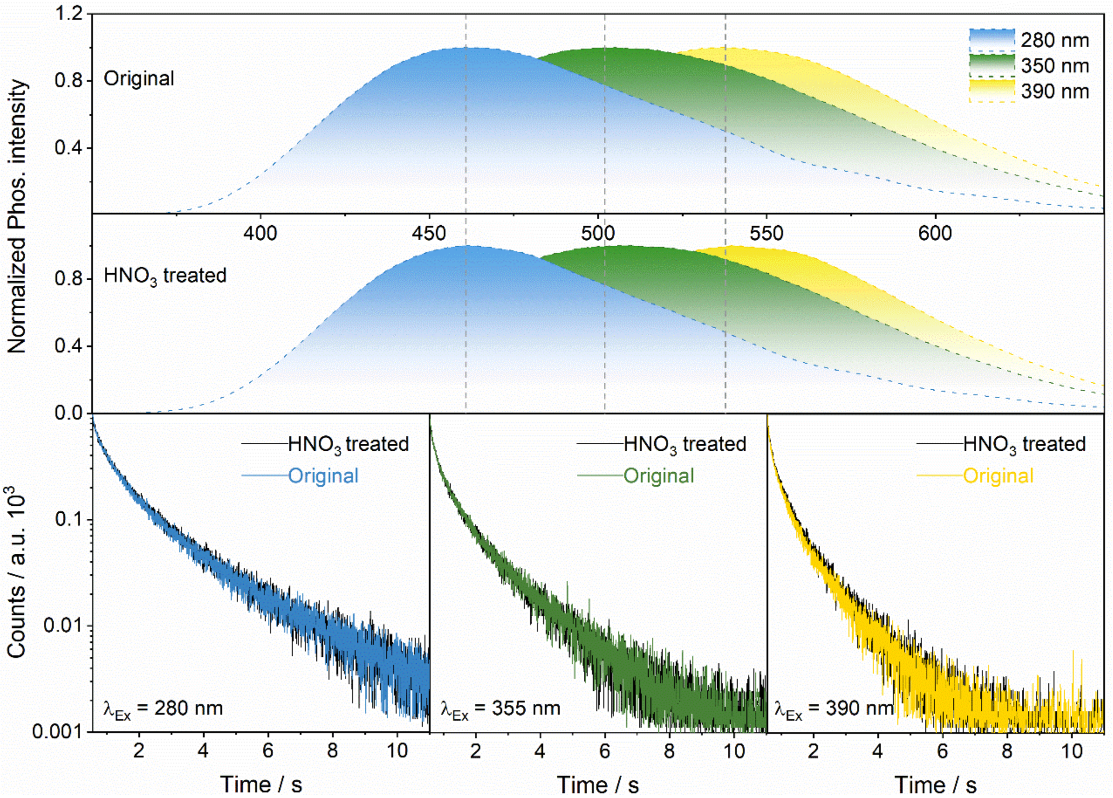

Supplementary Figure 10. Normalized phosphorescence spectra (delay time: 40 ms) and phosphorescence lifetime of untreated and HNO_3-saturated AlF_3 (excited by 280, 350 and 390 nm, respectively).

Both AlF_3 were calcinated from $AlF_3 \cdot 3H_2O$. One of the calcinated samples was saturated at HNO_3 for 90 min. After distilled water washing for 10 min, suction filtration and freeze drying, the AlF_3 powder was obtained. The emission profiles and lifetimes for HNO_3 treated AlF_3 were similar to the untreated sample. Therefore, the luminescence of AlF_3 was exactly originated from intrinsic emission, not the impurities of lanthanides or transition metal ions.

S3.2 Afterglow of α-AlF_3

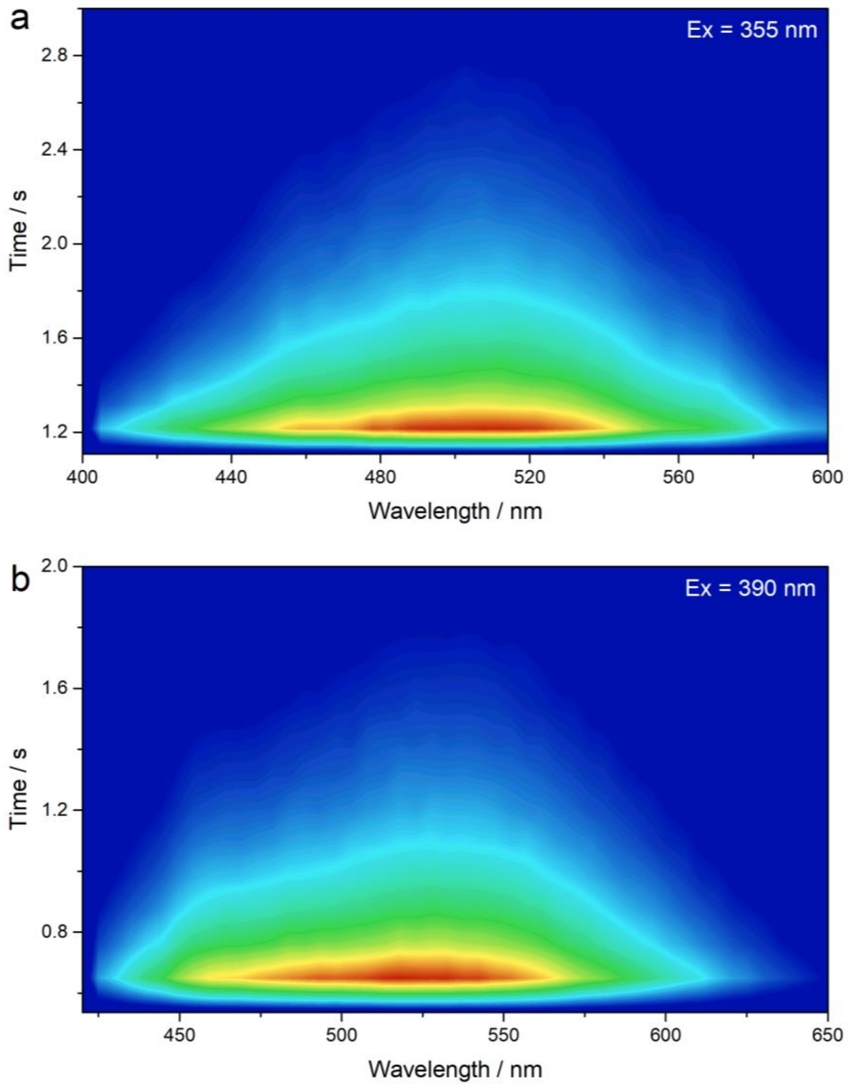

Supplementary Figure 11. Time-resolved emission spectra (TRES) of AlF_3 excited by 355 nm **(a)** and 390 nm **(b)**, respectively.

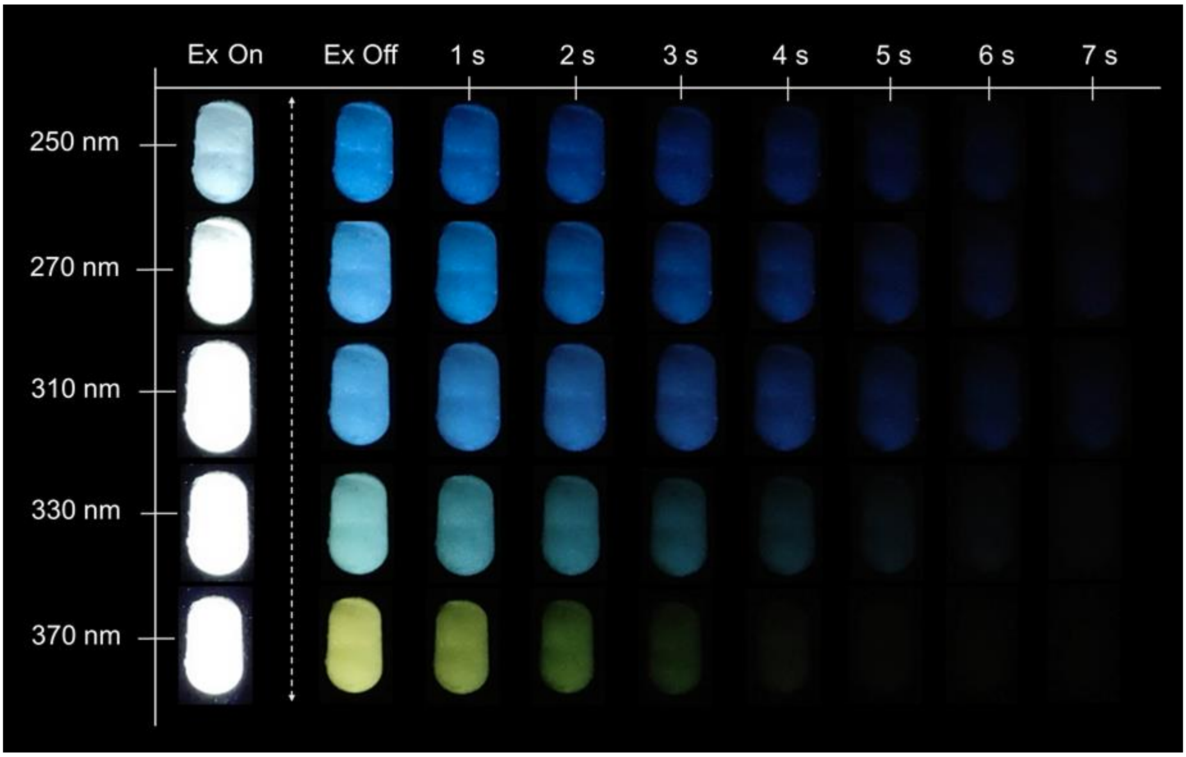

Supplementary Figure 12. Afterglow photographs of AlF_3 taken under different excitation (250, 270, 310, 330, and 370 nm) off.

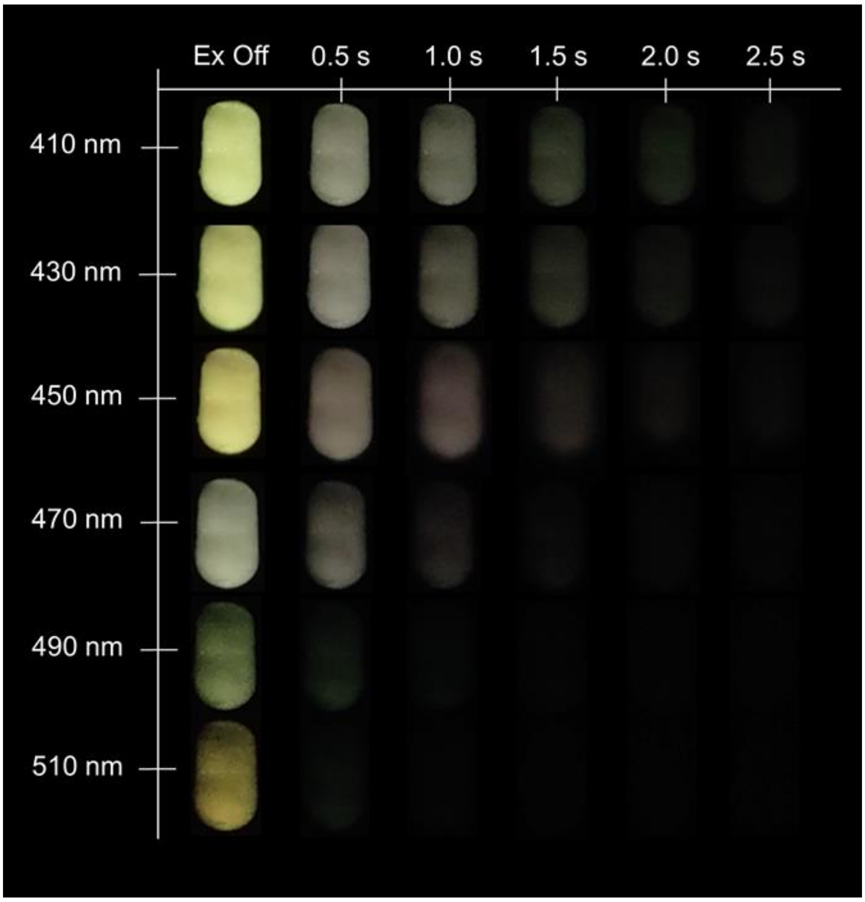

Supplementary Figure 13. Afterglow photographs of AlF_3 taken under different excitation (410-510 nm) off.

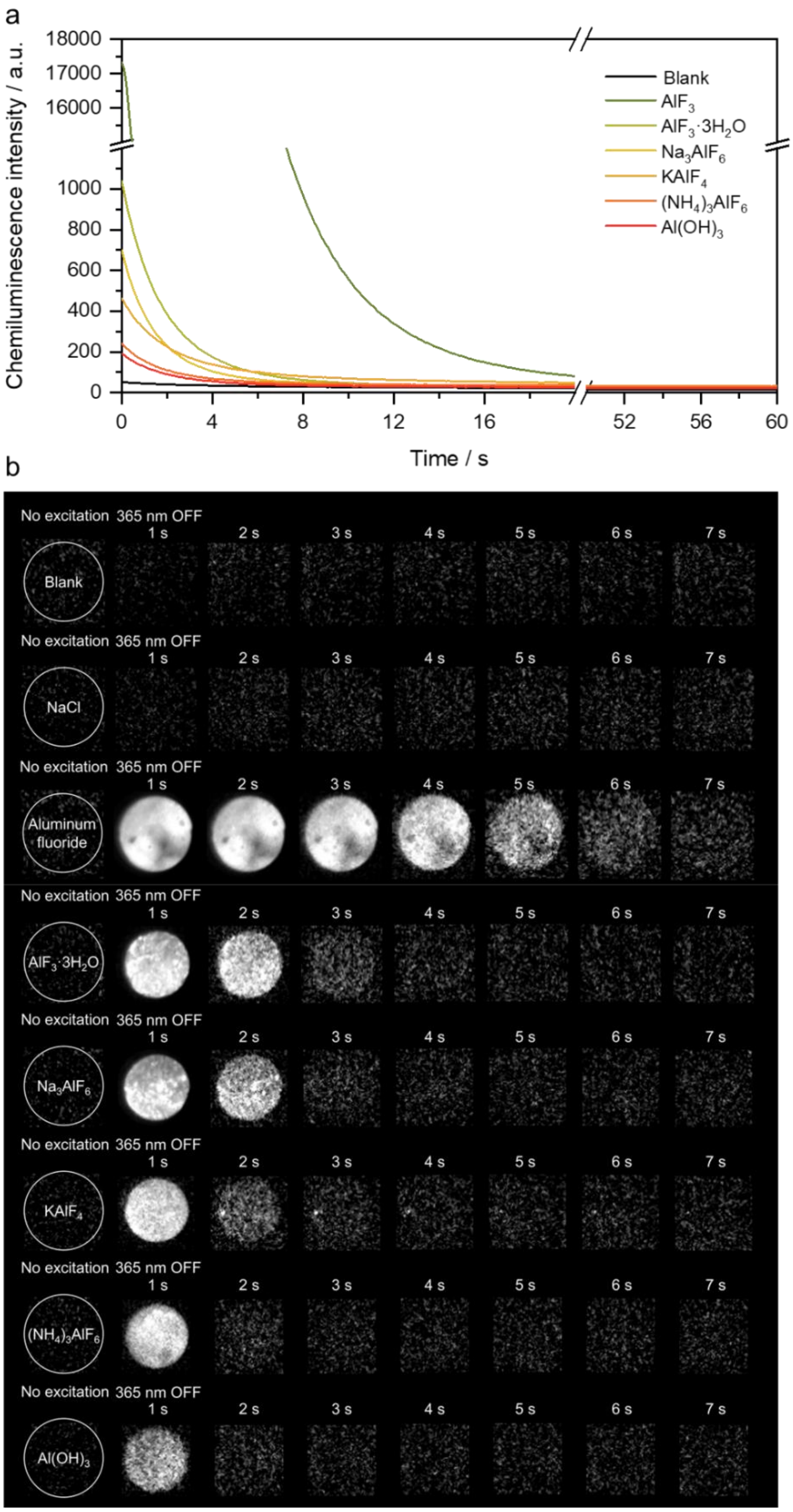

Supplementary Figure 14. Chemiluminescence profiles **(a)** and images **(b)** of the octahedral derivatives.

Azure C300 was implemented for chemiluminescence imaging, which contains a sensitive cold CCD detector. The detecting condition was optimized as follows: 30 s of

UV 365 nm radiation; 1 s interval between removal of UV 365 nm and staring detection; 1 s exposure time with normal sensitivity mode. The time courses of the decayed phosphorescence were taken under **MULTIPLE** mode with 1 s exposure. MPI-E chemiluminescence (CL) analyzer was explored to evaluate the phosphorescence intensity of the samples after turning off of the excitation (UV 365 nm). Specifically, no grating was equipped with the CL analyzer, and total luminescence was therefore collected. The detecting condition was optimized as follows: 30 s of UV 365 nm radiation, 1 s interval between UV turning off and staring detection. Each sample was tested under same condition at room temperature. For such measurements, we could further confirm the long-lived phosphorescence (no wavelength information, only total luminescence intensity) through exactly excitation ceasing.

S3.3 Additional photophysical data of α-AlF_3

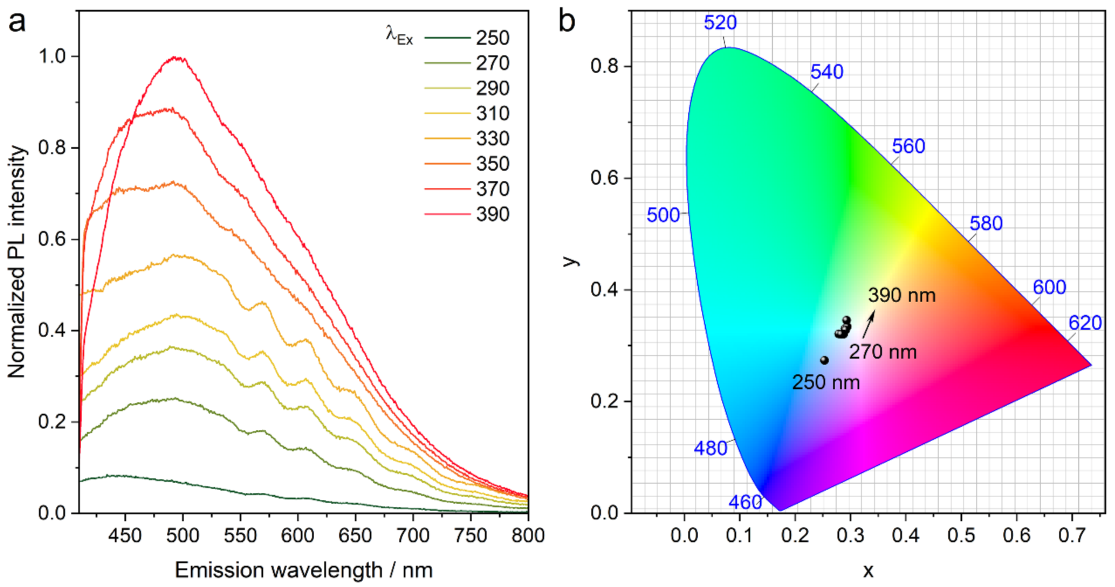

Supplementary Figure 15. Normalized PL spectra **(a)** and corresponding Commission Internationale de l'Eclairage (CIE) coordinate **(b)** of AlF_3 under different excitation (250 to 390 nm).

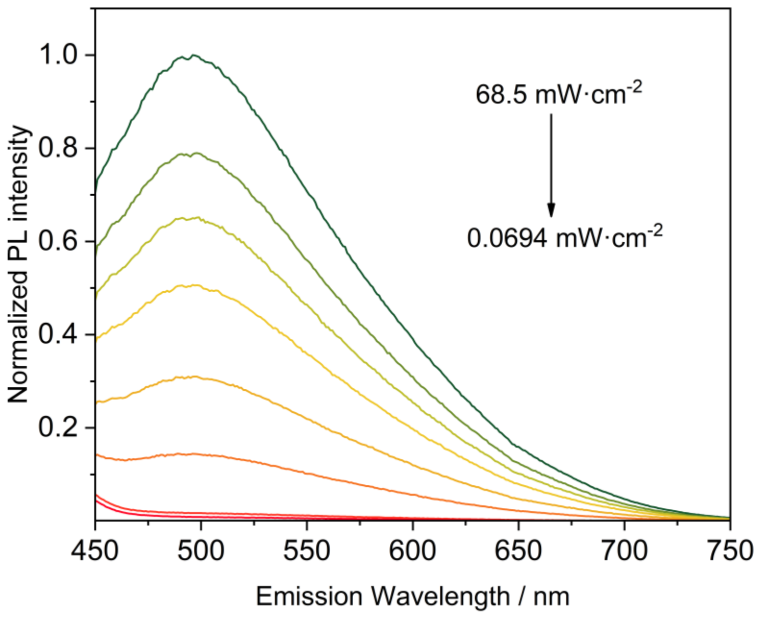

Supplementary Figure 16. Excitation power density-dependent PL spectra of AlF_3 (λ_{ex} = 405 nm)

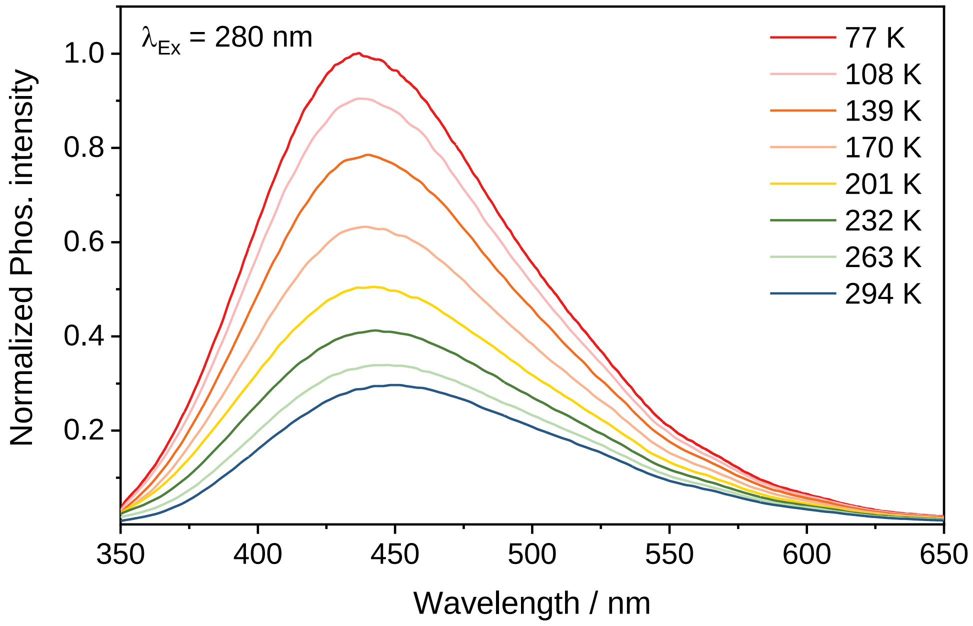

Supplementary Figure 17. Temperature-dependent phosphorescence spectra of AlF_3 (λex = 280 nm, delay time of 40 ms)

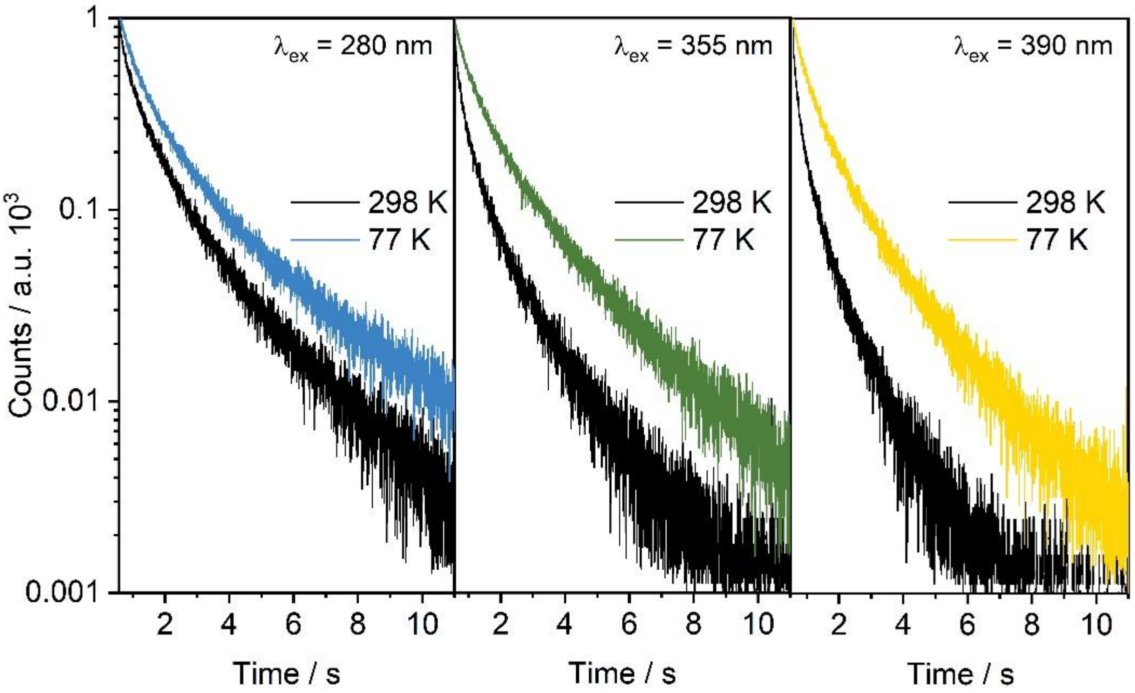

Supplementary Figure 18. Phosphorescence lifetime of AlF_3 at room temperature and 77 K condition. (excited by 280, 350 and 390 nm, respectively).

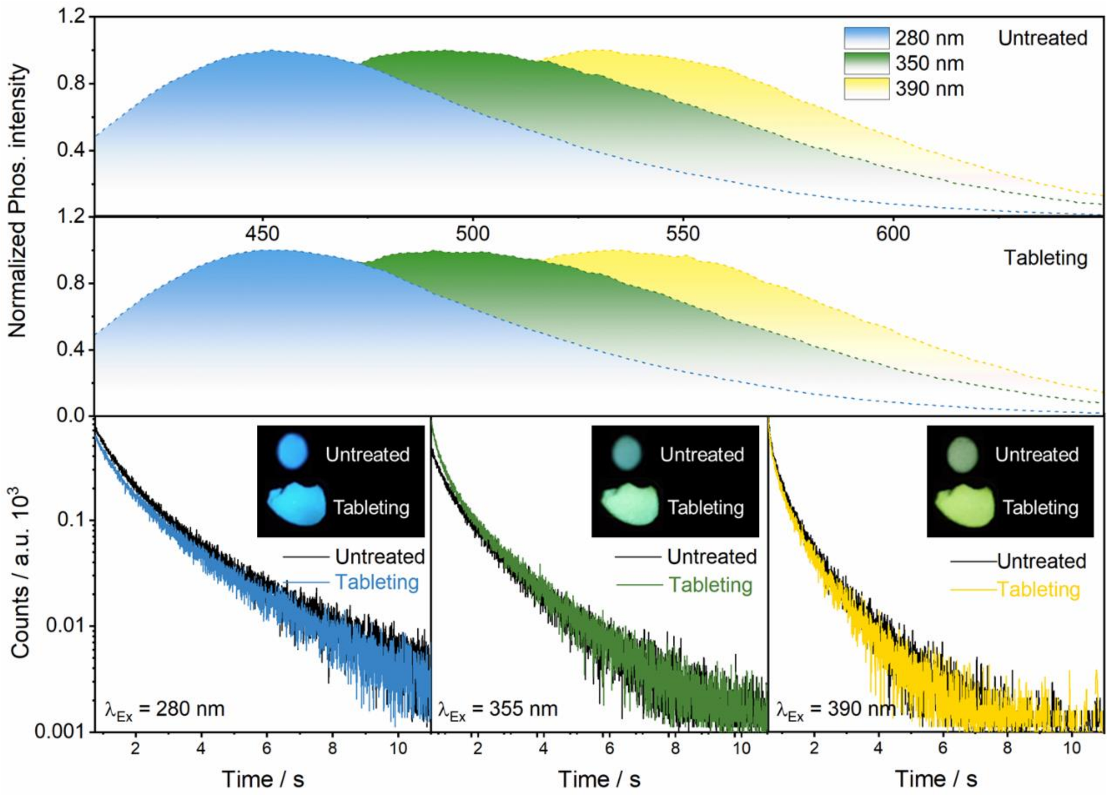

Supplementary Figure 19. Normalized phosphorescence spectra and phosphorescence lifetime of AlF_3 before and after tableting, respectively.

Section S4. The Octahedral unit derivatives

S4.1 Structures

The crystalline structures of aluminum fluoride, other octahedral derivatives (potassium fluoroaluminate, ammonium hexafluoroaluminate, aluminum chloride, indium chloride and cadmium chloride, Supplementary Table 6) and aluminum bromide were obtained from Inorganic Crystal Structure Database (ICSD, https://icsd.products.fiz-karlsruhe.de/), Crystallography Open Database (COD, http://www.crystallography.net/cod/) and Materials Project (https://www.materialsproject.org/).

Supplementary Table 7. Crystalline information of the substance used in this work.

No.	Name	Chemical Structure	Database	ID	Space Group
1	Aluminum fluoride	AlF_3	ICSD	68826	R3c
2	Potassium fluoroaluminate	$KAlF_4$	ICSD	60524	P4/mbm
3	Ammonium hexafluoroaluminate	$(NH_4)_3AlF_6$	COD	1010577	F-43m
4	Aluminum Chloride	$AlCl_3$	COD	1535669	C2/m
5	Aluminum Bromide	$AlBr_3$	COD	1535698	P21/c
6	Indium chloride	$InCl_3$	Materials Project	mp-862983	$P6_3/mmc$
7	Cadmium chloride	$CdCl_2$	Materials Project	mp-695850	C2/m

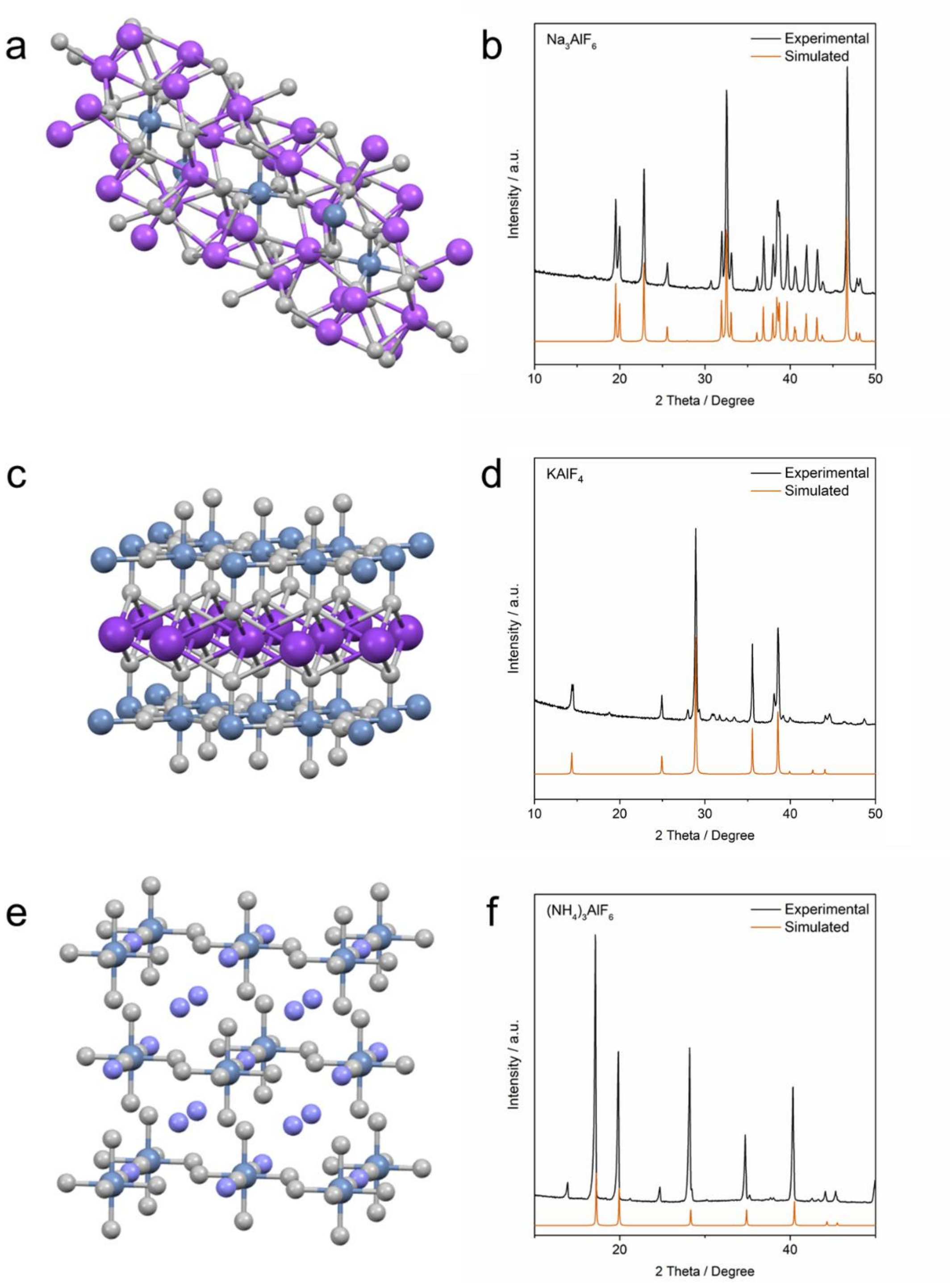

Supplementary Figure 20. Crystalline data of the derivatives featuring AlF_6 octahedra. **a-b**, Na_3AlF_6. **c-d**, $KAlF_4$. **e-f**, $(NH_4)_3AlF_6$.

Na_3AlF_6, $KAlF_4$, $(NH_4)_3AlF_6$ all belong to AlF_6 derivatives and they all exhibit phosphorescence. Meanwhile, crystalline structure of $KAlF_4$ and $(NH_4)_3AlF_6$ demonstrate that they possess 2D and 0D perovskite-like structures, respectively. Hence, $KAlF_4$ and $(NH_4)_3AlF_6$ are further discussed in octahedral unit luminescence.

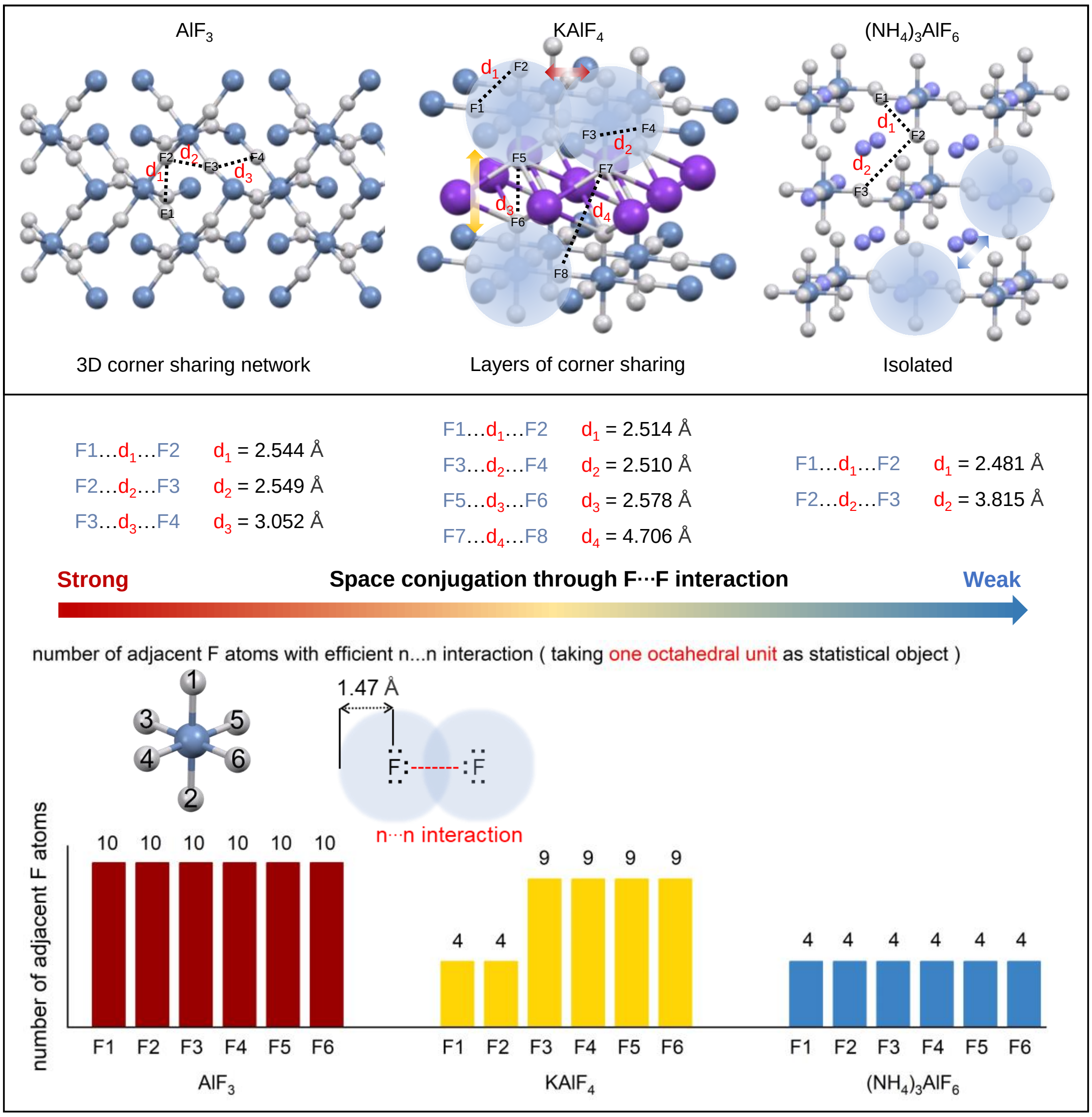

Supplementary Figure 21. Schematic illustration of AlF_6 octahedra space conjugation with diverse dimension (3D network, layers of corner sharing and isolated). Crystalline structure of AlF_3, $KAlF_4$, $(NH_4)_3AlF_6$ are from ISCD 68826, ICSD 60524 and COD 1010577, respectively.

S4.2 Photophysical properties

Supplementary Table 8. Summary of the emission properties of AlF_6 derivatives.

Chemical Structure	λ_{ex} = 280 nm		λ_{ex} = 355 nm	
	$\lambda_{em,\ P}$ / nm	τ_P / s	$\lambda_{em,\ P}$ / nm	τ_P / s
$AlF_3 \cdot 3H_2O$	450	0.686	506	0.356
Na_3AlF_6	452	0.345	484	0.307
$KAlF_4$	480	0.571	502	0.400
$(NH_4)_3AlF_6$	446	0.268	496	0.321

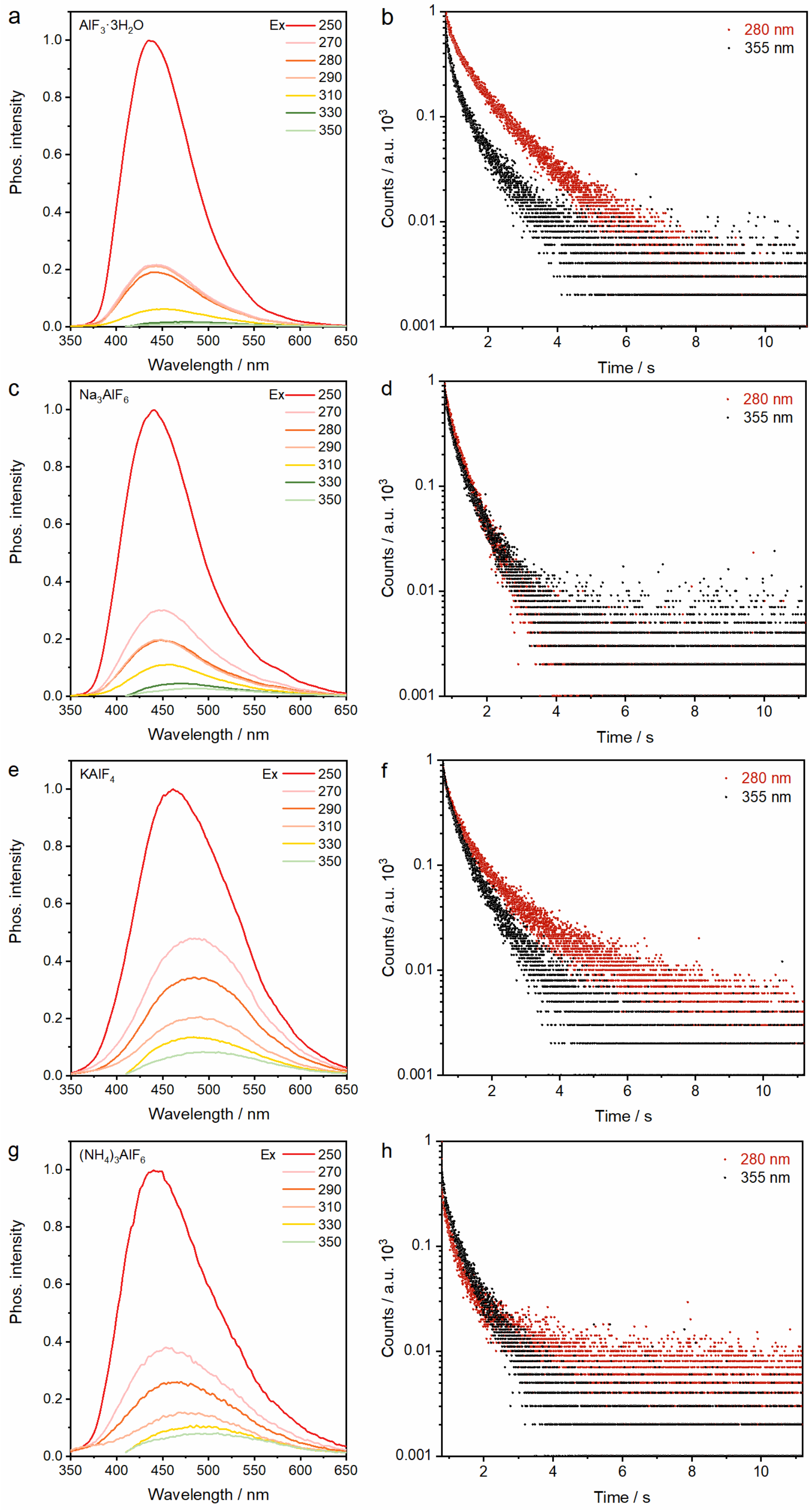

Supplementary Figure 22. The excitation-dependent phosphorescence spectra (delay time: 40 ms) and phosphorescent lifetime of AlF_3 derivatives. **a-b**, $AlF_3 \cdot H_2O$. **c-d**, Na_3AlF_6. **e-f**, $KAlF_4$. **g-h**, $(NH_4)_3AlF_6$.

Supplementary Table 9. Summary of the emission properties of other octahedral derivatives.

Chemical Structure	λ_{ex} = 280 nm		Chemical Structure	λ_{ex} = 280 nm	
	λ_{em} nm	τ_P		λ_{em} nm	τ_P
$AlCl_3$	486	0.143 s	$AlBr_3$	496	0.610 ms
Al_2O_3	450	0.311 s	$Al(OH)_3$	450	0.297 s
Ga_2O_3	500	15.3 ms	$InCl_3$	468	68.7 ms
$CdCl_2$	460	9.00 µs			

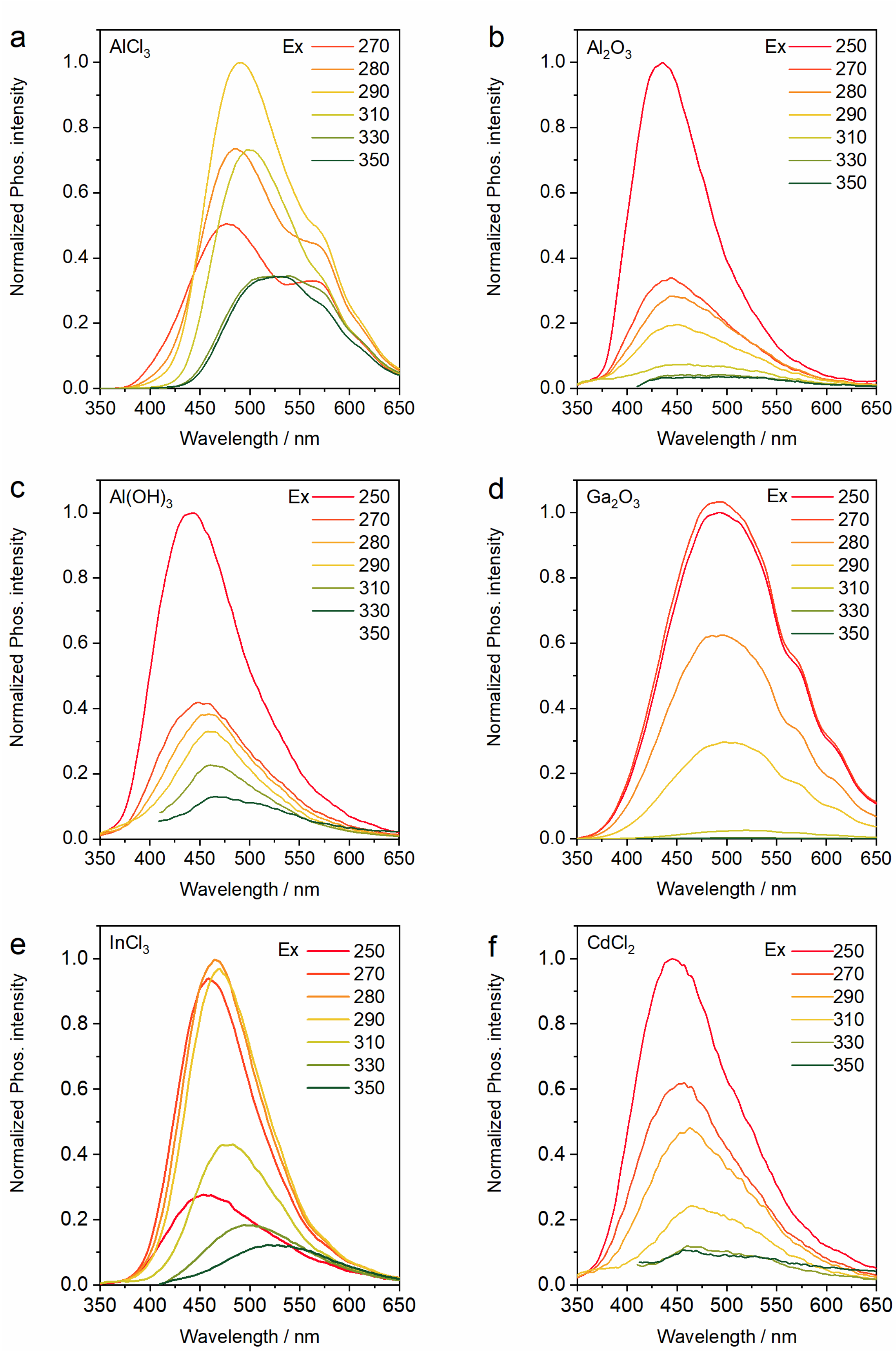

Supplementary Figure 23. The excitation-dependent phosphorescence spectra (delay time: 40 ms for $AlCl_3$, $Al(OH)_3$, Al_2O_3, Ga_2O_3 and $InCl_3$, 20 ms for $CdCl_2$). **a**, $AlCl_3$. **b**, Al_2O_3. **c**, $Al(OH)_3$. **d**, Ga_2O_3. **e**, $InCl_3$. **f**, $CdCl_2$.

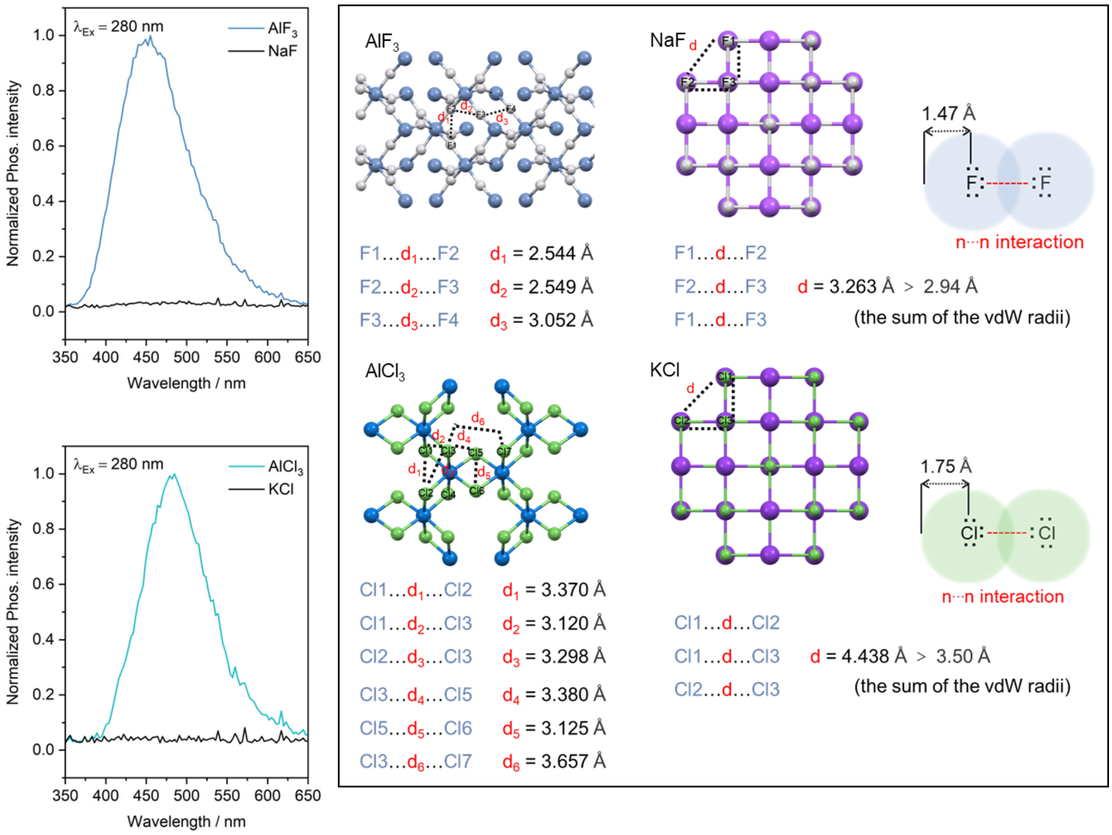

Supplementary Figure 24. Phosphorescence spectra of AlF_3, NaF, $AlCl_3$ and KCl excited at 280 nm and their structural analysis.

S4.3 Afterglow images

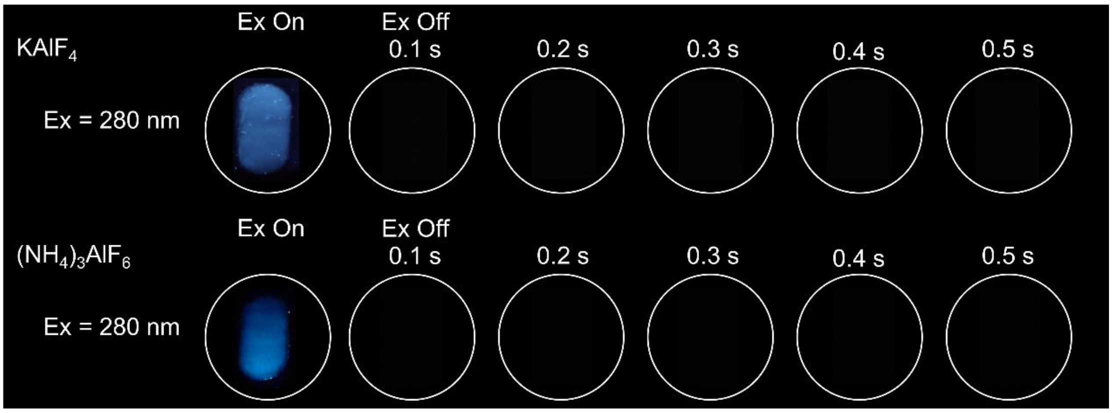

Supplementary Figure 25. Afterglow images of $KAlF_4$ and $(NH_4)_3AlF_6$ (altering dimension) at 298 K.

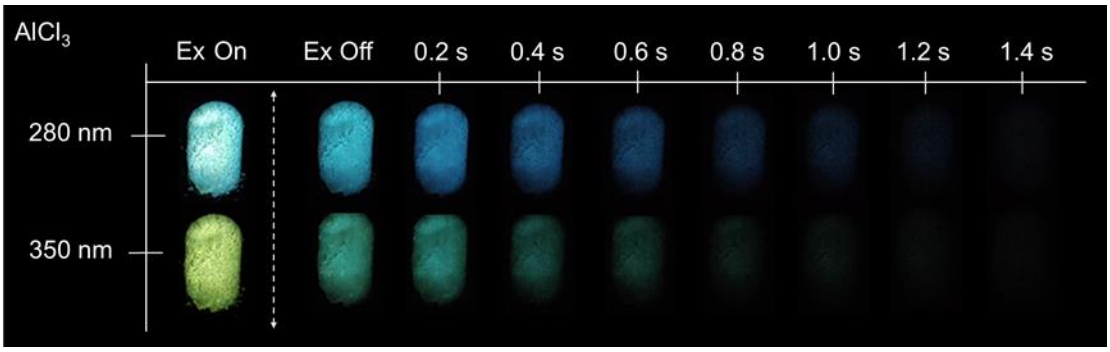

Supplementary Figure 26. Afterglow images of $AlCl_3$ (altering ligand) at 298 K.

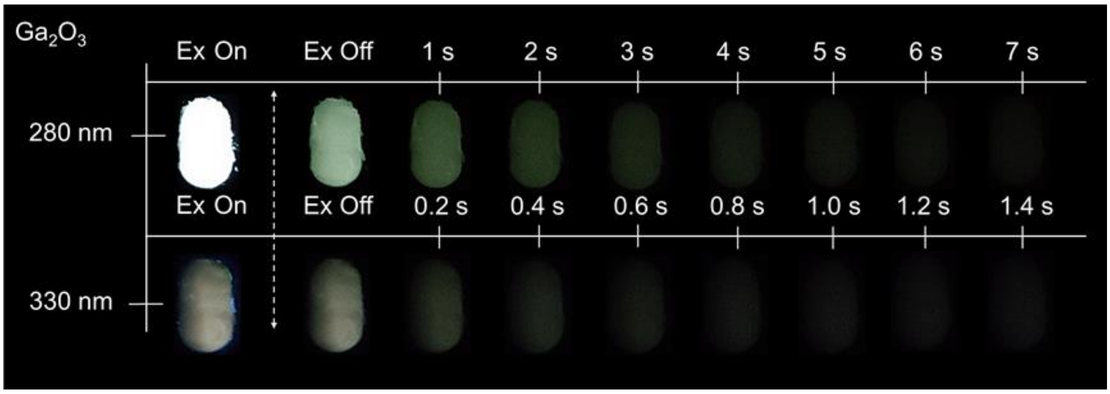

Supplementary Figure 27. Afterglow images of Ga_2O_3 (altering center) at 298 K.

Section S5. Theoretical calculation

S5.1 Theoretical calculation of AlF_3

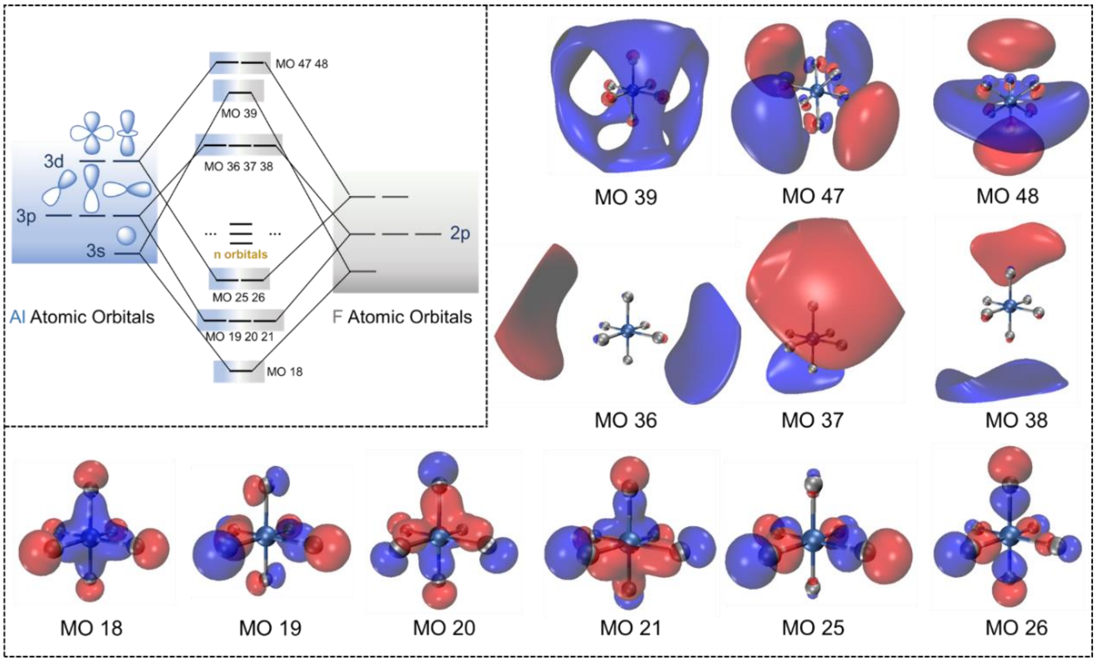

Supplementary Figure 28. The schematic diagram of $[AlF_6]^{3-}$ orbital distribution based on ligand field theory and calculated isosurfaces of the molecular orbitals (isovalue = 0.01~0.02) corresponding.

In one unit of AlF_6 octahedron, central Al contributed one 3*s*, three 3*p* and two 3*d* orbitals to participate in hexa-coordination. The other constitution of bonds was from one *p* orbital of six F, respectively. The ascription of molecule orbitals from bonded and unbonded electron was described in Supplementary Fig. 20.

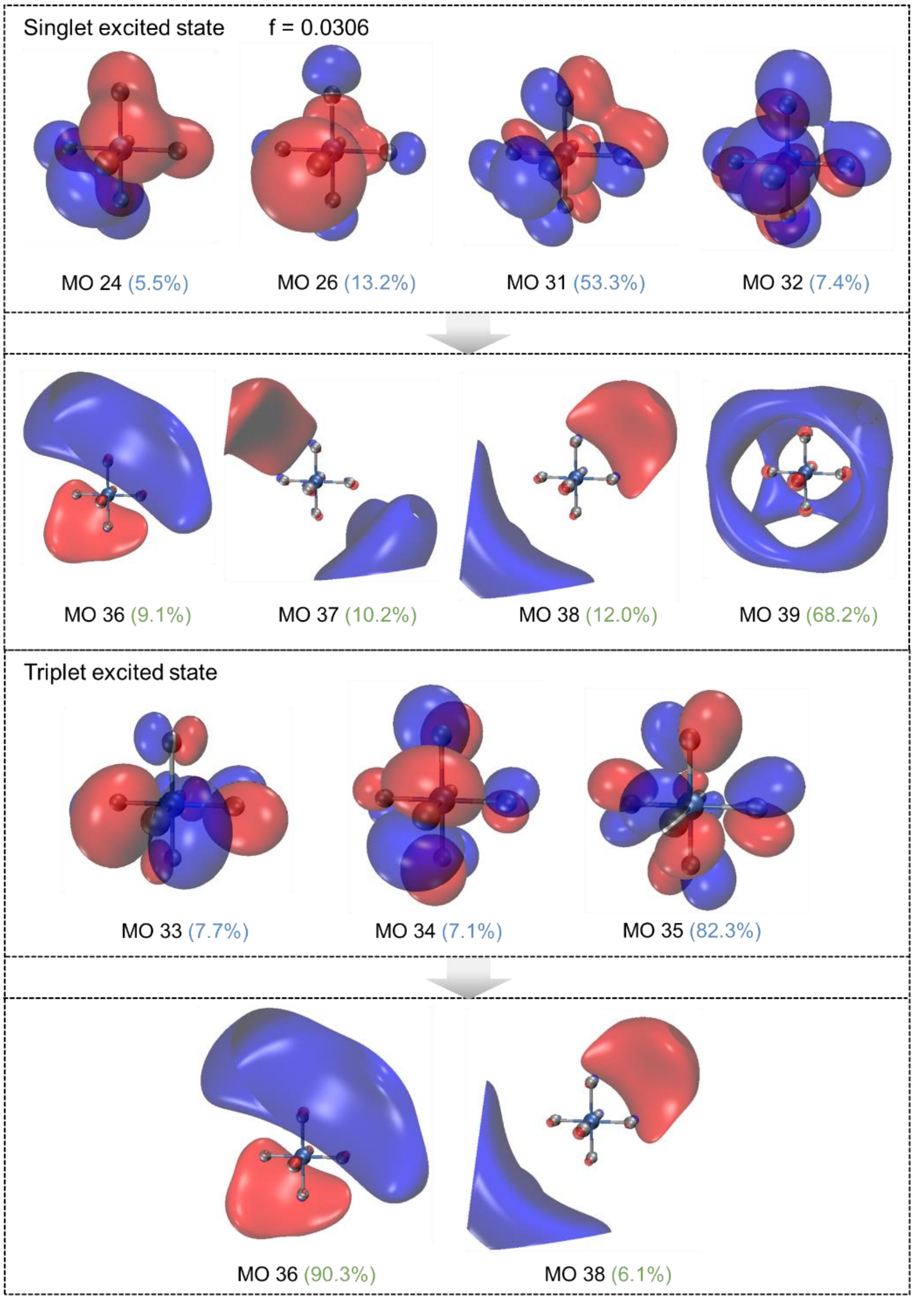

Supplementary Figure 29. Calculated isosurfaces of $[AlF_6]^{3-}$ dominant molecular orbitals (isovalue = 0.015~0.055) for singlet excited state with maximum oscillator strength and lowest triplet excited state and their contribution to hole and electron distribution (%).

Note: **blue number** refers to the contribution of Hole, **green number** refers to the contribution of Electron.

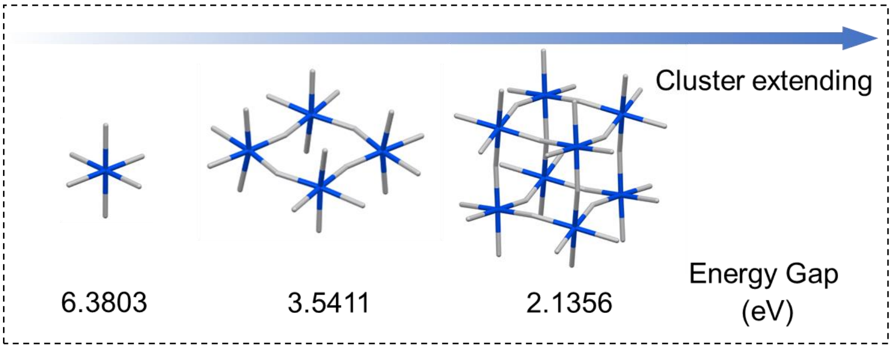

Supplementary Figure 30. Schematic illustration of the AlF_6 clusters and the corresponding calculated energy gap (isolated, layered and cubic corner sharing AlF_6 octahedra).

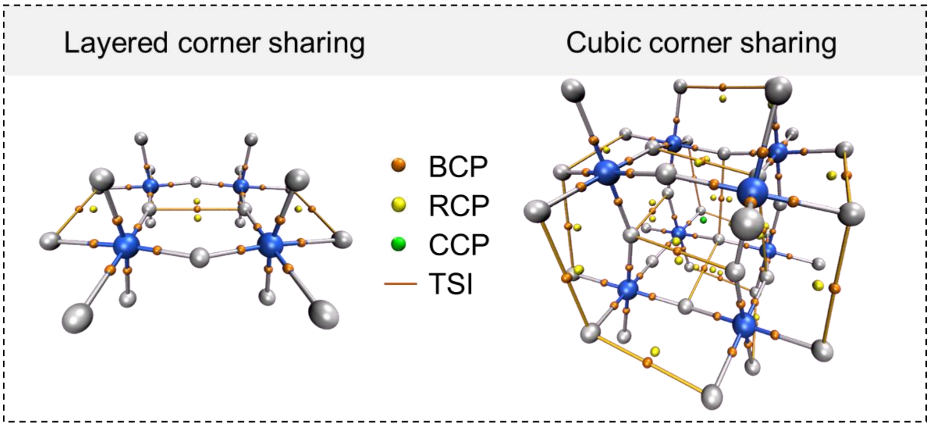

Supplementary Figure 31. AIM analysis of layered and cubic corner sharing AlF_6 octahedra. The bond (BCP), ring (RCP) and cage (CCP) critical points represent the extreme points of electron density on the bond paths, centers of rings, and enclosed space formed by rings, respectively. Through space interaction (TSI) paths were indicated by orange dash lines.

S5.2 Theoretical calculation of octahedral unit derivatives

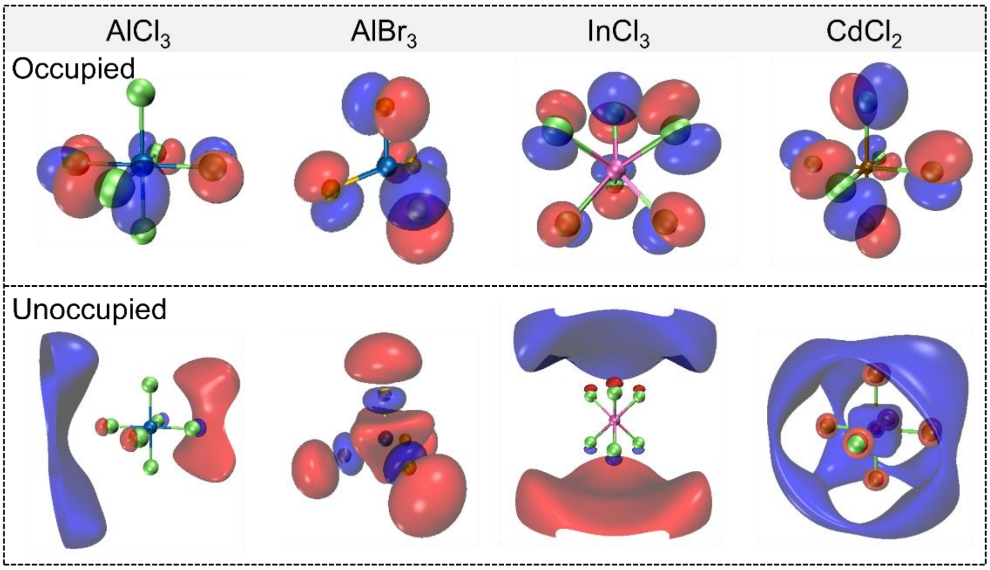

Supplementary Figure 32. The TD-DFT calculated isosurfaces of occupied and unoccupied orbitals related to $T_1 \rightarrow S_0$ transition of $AlCl_3$, $AlBr_3$, $InCl_3$, and $CdCl_2$ octahedral unit, respectively.

To further confirm the calculation results, single octahedral unit of $AlCl_3$, $InCl_3$ and $CdCl_2$ and the tetrahedral unit of $AlBr_3$ were further investigated with TDDFT. For the $T_1 \rightarrow S_0$ transitions, the HOMO of their octahedra all comprise *p* orbitals of chloride, while the LUMO of $AlCl_3$ and $InCl_3$ consist of *p* orbitals from metal and chloride, which is preferred for stabilization of the excited triplet state, thus resulting in notable phosphorescence. Such results were similar to that of AlF_3 and agreed well with experimental results (Fig. 3d, f). On the contrary, the LUMO of $AlBr_3$ and $CdCl_2$ were contributed by the Al *s* orbital, which resulted in reduced parity-forbidden effect as compared with AlF_3, and thus relatively weak phosphorescence.

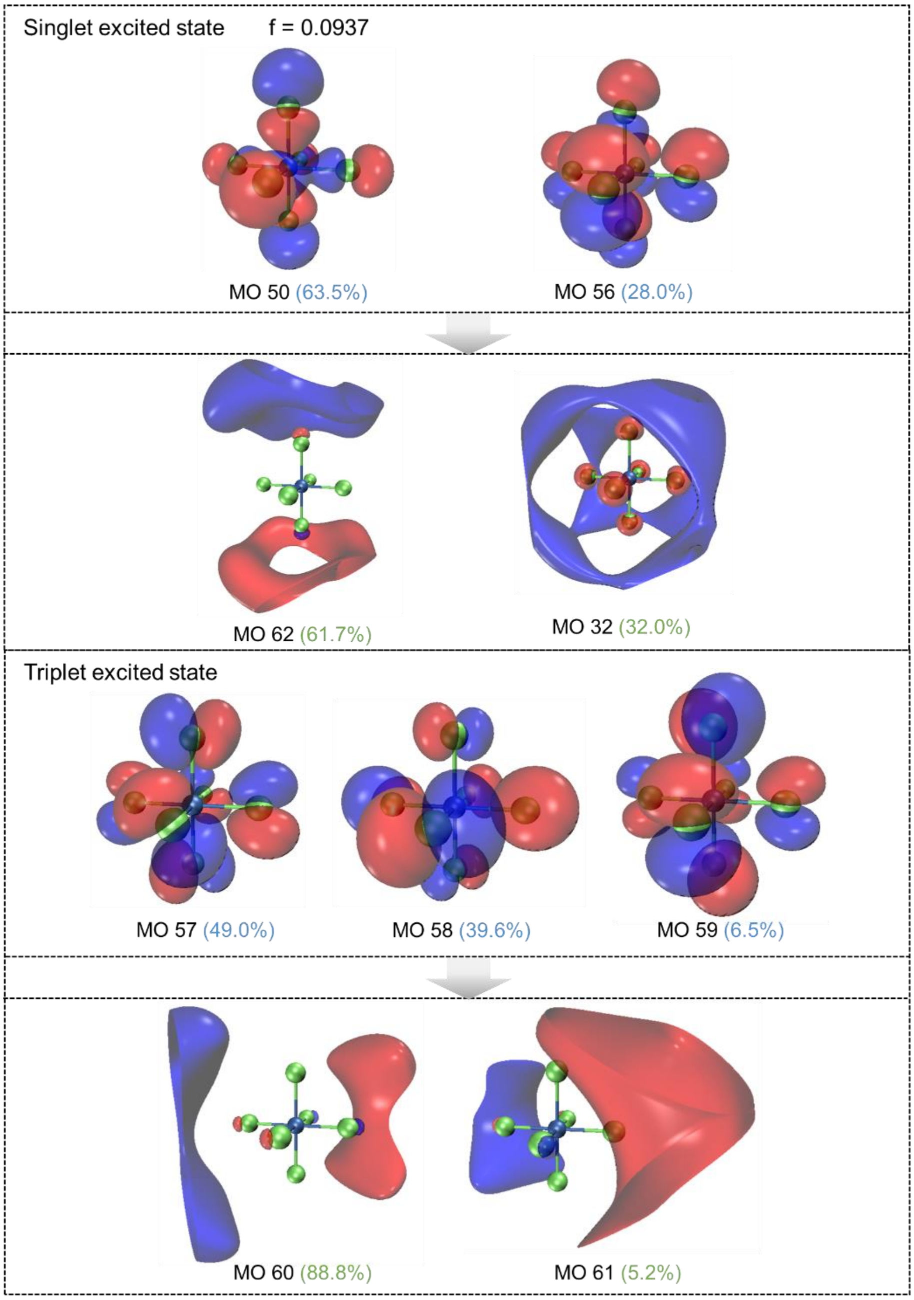

Supplementary Figure 33. Calculated isosurfaces of $[AlCl_6]^{3-}$ dominant molecular orbitals (isovalue = 0.015~0.055) for singlet excited state with maximum oscillator strength and lowest triplet excited state and their contribution to hole and electron distribution (%).

Note: **blue number** refers to the contribution of Hole, **green number** refers to the contribution of Electron.

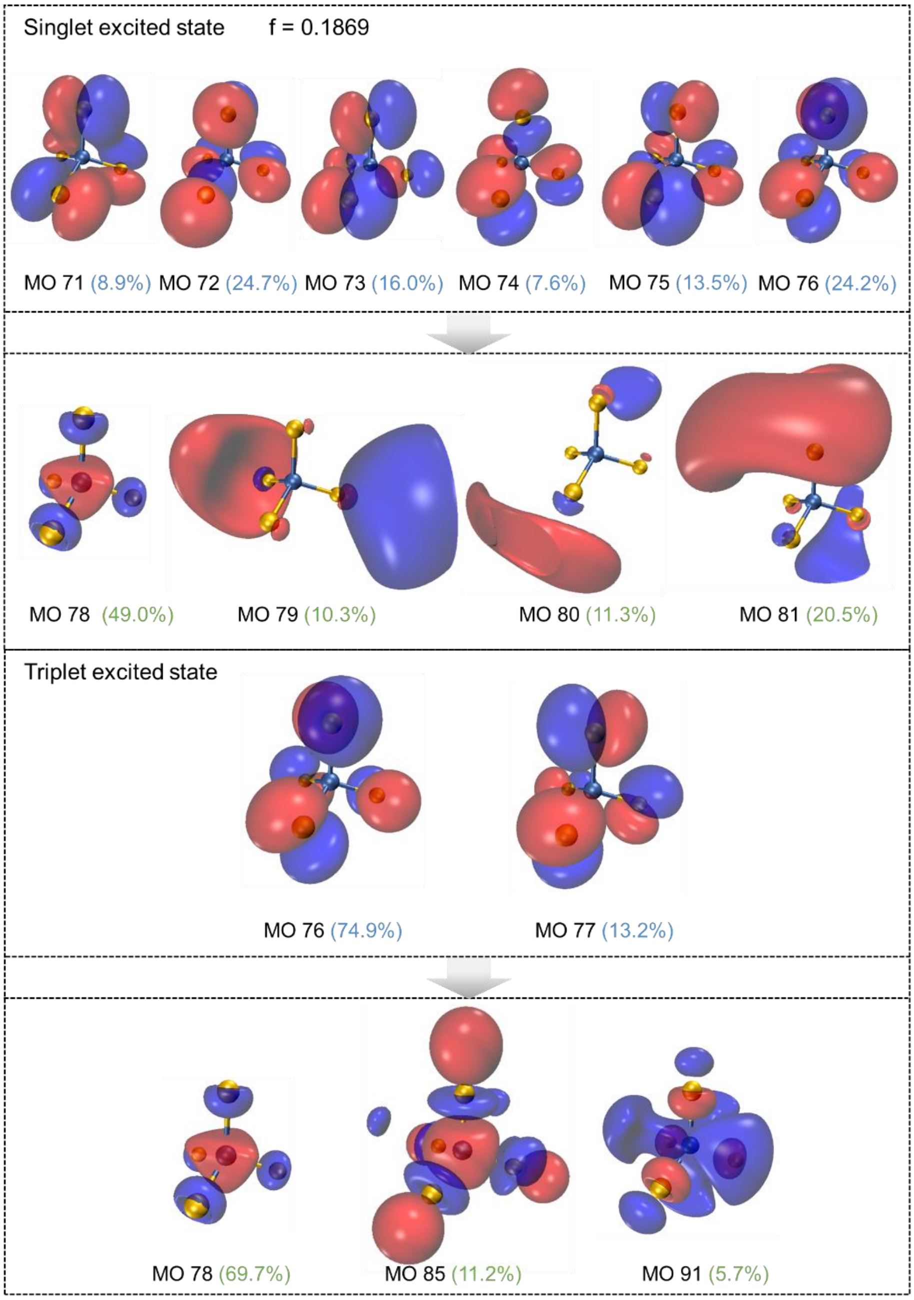

Supplementary Figure 34. Calculated isosurfaces of $[AlBr_4]^-$ dominant molecular orbitals (isovalue = 0.015~0.055) for singlet excited state with maximum oscillator strength and lowest triplet excited state and their contribution to hole and electron distribution (%).

Note: **blue number** refers to the contribution of Hole, **green number** refers to the contribution of Electron.

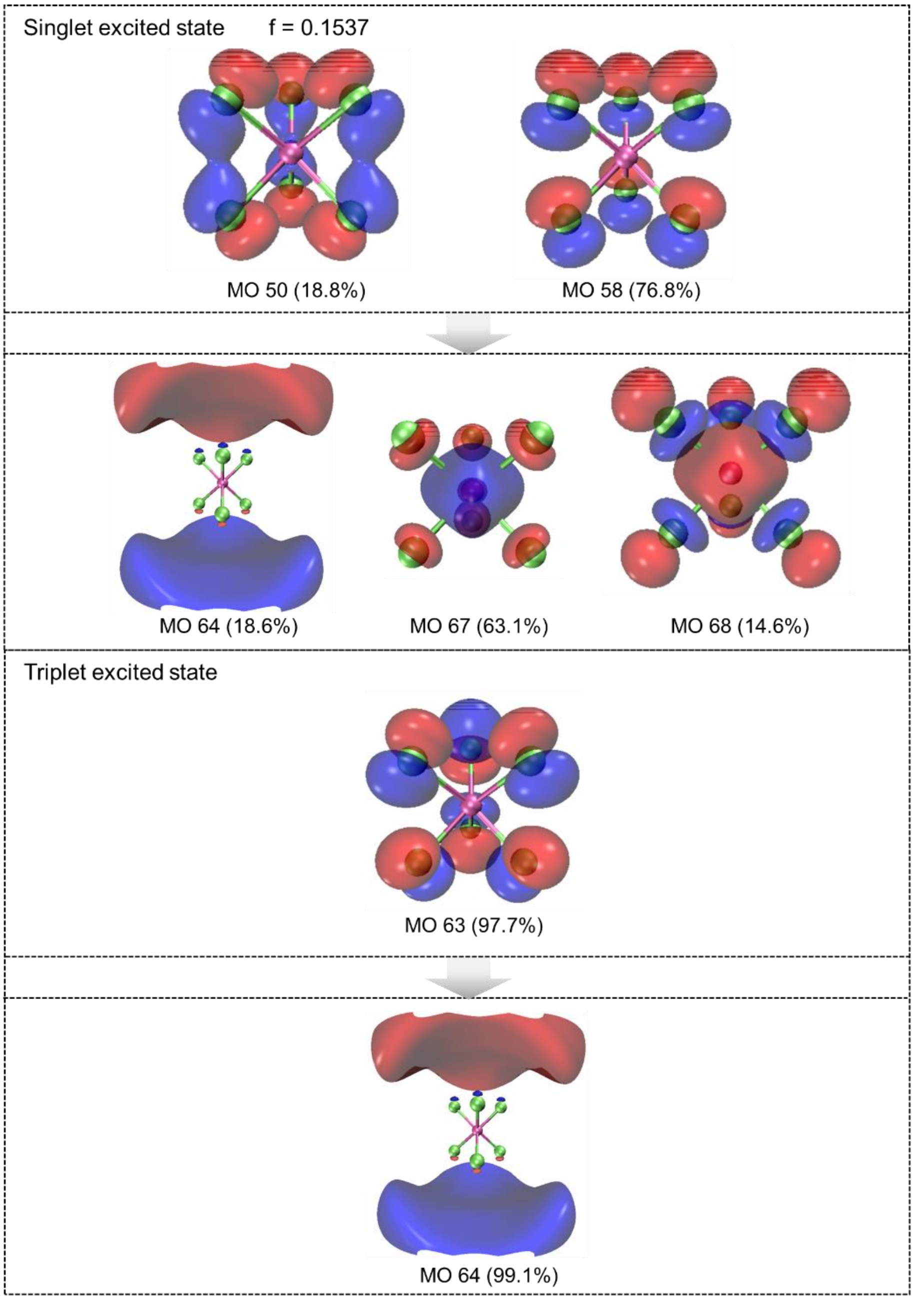

Supplementary Figure 35. Calculated isosurfaces of $[InCl_6]^{3-}$ dominant molecular orbitals (isovalue = 0.015~0.055) for singlet excited state with maximum oscillator strength and lowest triplet excited state and their contribution to hole and electron distribution (%).

Note: **blue number** refers to the contribution of Hole, **green number** refers to the contribution of Electron.

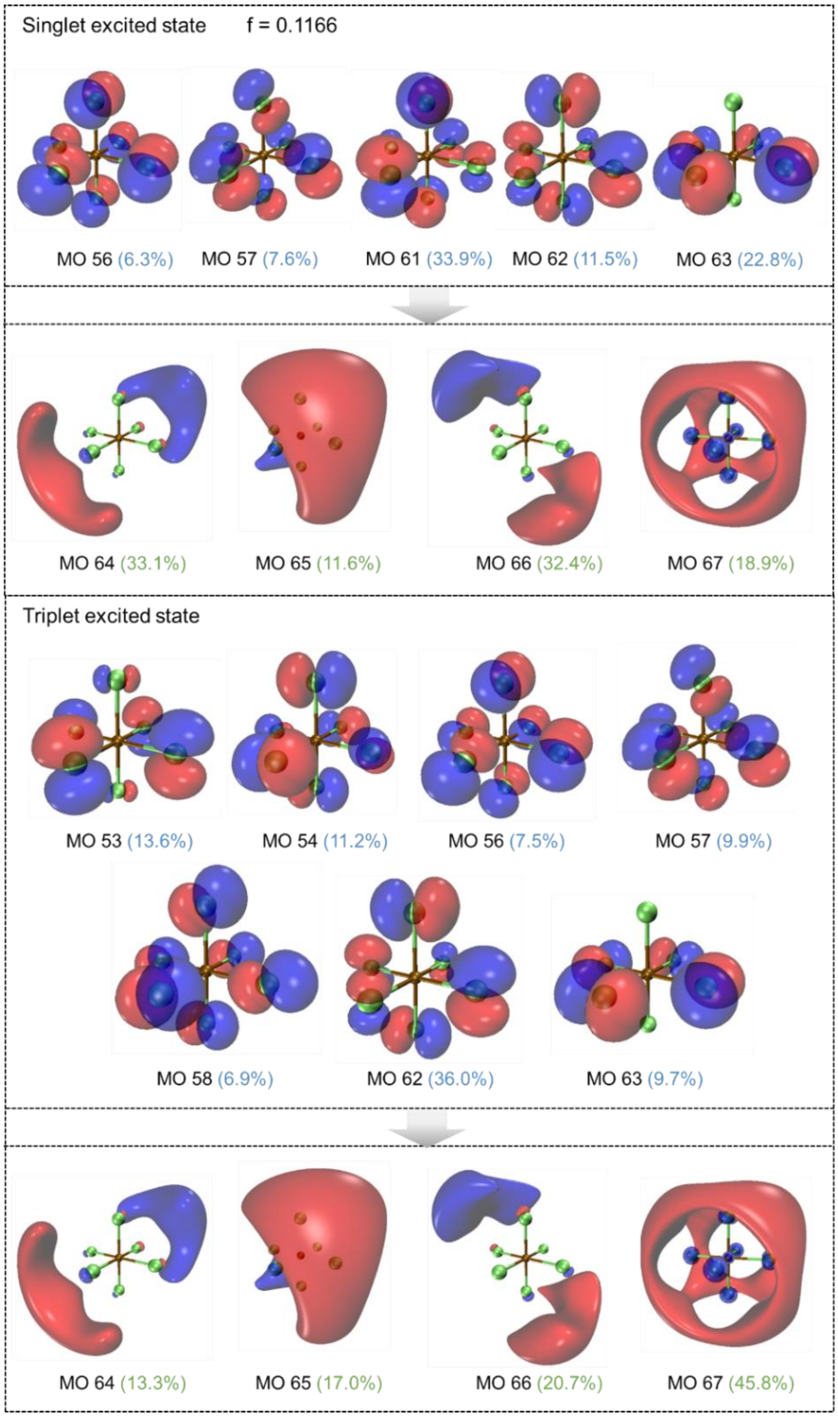

Supplementary Figure 36. Calculated isosurfaces of $[CdCl_6]^{4-}$ dominant molecular orbitals (isovalue = 0.015~0.055) for singlet excited state with maximum oscillator strength and lowest triplet excited state and their contribution to hole and electron distribution (%).

Note: **blue number** refers to the contribution of Hole, **green number** refers to the contribution of Electron.

Supplementary References

(1) Matsuzawa, T.; Aoki, Y.; Takeuchi, N.; Murayama, Y. *J. Electrochem. Soc.* **1996**, *143*, 2670-2673.

(2) Wang, X.-J.; Jia, D.; Yen, W. M. *J. Lumin.* **2003**, *102-103*, 34-37.

(3) Asatryan, G. R.; Kulinkin, A. B.; Feofilov, S. P.; Hovhannesyan, K. L.; Petrosyan, A. G. *Phys. Solid State* **2017**, *59*, 487-489.

(4) Jia, D.; Yen, W. M. *J. Lumin.* **2003**, *101*, 115-121.

(5) Savchuk, V. A.; Korzun, B. V.; Sobolev, N. A.; Makovetskaya, L. A. *Semiconductors* **1997**, *31*, 315-318.

(6) Xu, J.; Cherepy, N. J.; Ueda, J.; Tanabe, S. *Mater. Lett.* **2017**, *206*, 175-177.

(7) Lin, R.; Zheng, W.; Chen, L.; Zhu, Y.; Xu, M.; Ouyang, X.; Huang, F. *Nat. Commun.* **2020**, *11*, 4351.

(8) Yu, Z. Q.; Chang, D.; Li, C.; Zhang, N.; Feng, Y. Y.; Dai, Y. Y. *J. Mater. Res.* **2001**, *16*, 1890-1893.

(9) Gaponenko, N. V.; Molchan, I. S.; Gaponenko, S. V.; Mudryi, A. V.; Lyutich, A. A.; Misiewicz, J.; Kudrawiec, R. *J. Appl. Spectrosc.* **2003**, *70*, 59-64.

(10) Joseph, J.; Anappara, A. A. *ChemistrySelect* **2017**, *2*, 4058-4062.

(11) Ding, W.; Peng, L.; Yun, D.; Gao, S.; Duan, R.; Gu, Y.; Wang, C.; Li, W.; Zeng, X.; Sun, F. *ACS Appl. Bio Mater.* **2020**, *3*, 1712-1721.

(12) Wei, M.; Zhong, H.; Zhou, J.; Liu, W.; Xi, W.; Xu, P.; Qiu, Q.; Qian, Z.; Feng, H. *Talanta* **2020**, *219*, 121298.

(13) Burrows, H. D.; Fernandes, M.; Seixas de Melo, J.; Monkman, A. P.; Navaratnam, S. *J. Am. Chem. Soc.* **2003**, *125*, 15310-15311.

(14) Nandi, S.; Mondal, A.; Reinsch, H.; Biswas, S. *Inorg. Chim. Acta* **2019**, *497*, 119078.

(15) Nandi, S.; Reinsch, H.; Biswas, S. *Microporous Mesoporous Mater.* **2020**, *293*, 109790.

(16) Liu, F.; Yan, W.; Chuang, Y.-J.; Zhen, Z.; Xie, J.; Pan, Z. *Sci. Rep.* **2013**, *3*, 1554.

(17) Macfarlane, P. I.; Han, T. P. J.; Henderson, B.; Kaminskii, A. A. *Opt. Mater.* **1994**, *3*, 15-24.

(18) Pan, Z.; Lu, Y.-Y.; Liu, F. *Nat. Mater.* **2012**, *11*, 58-63.

(19) Sun, W.; Pang, R.; Li, H.; Li, D.; Jiang, L.; Zhang, S.; Fu, J.; Li, C. *J. Mater. Chem. C* **2017**, *5*, 1346-1355.

(20) Bessière, A.; Jacquart, S.; Priolkar, K.; Lecointre, A.; Viana, B.; Gourier, D. *Opt. Express* **2011**, *19*, 10131-10137.

(21) Jeong, I.-K.; Park, H. L.; Mho, S.-i. *Solid State Commun.* **1998**, *105*, 179-183.

(22) Heikenfeld, J.; Garter, M.; Lee, D. S.; Birkhahn, R.; Steckl, A. J. *Appl. Phys. Lett.* **1999**, *75*, 1189-1191.

(23) Miyata, T.; Nakatani, T.; Minami, T. *J. Lumin.* **2000**, *87-89*, 1183-1185.

(24) Dong, L.; Jia, R.; Xin, B.; Peng, B.; Zhang, Y. *Sci. Rep.* **2017**, *7*, 40160.

(25) Binet, L.; Gourier, D. *J. Phys. Chem. Solids* **1998**, *59*, 1241-1249.

(26) Pearton, S. J.; Yang, J.; Cary, P. H.; Ren, F.; Kim, J.; Tadjer, M. J.; Mastro, M. A. *Appl. Phys. Rev.* **2018**, *5*, 011301.

(27) Chen, T.; Tang, K. *Appl. Phys. Lett.* **2007**, *90*, 053104.

(28) Wang, T.; Farvid, S. S.; Abulikemu, M.; Radovanovic, P. V. *J. Am. Chem. Soc.* **2010**, *132*, 9250-9252.

(29) Huang, H.-L.; Huang, Y.-T.; Wang, S.-L. *Inorg. Chem.* **2016**, *55*, 6836-6838.

(30) Gross, U.; Rüdiger, S.; Kemnitz, E.; Brzezinka, K.-W.; Mukhopadhyay, S.; Bailey, C.; Wander, A.;

Harrison, N. *J. Phys. Chem. A* **2007**, *111*, 5813-5819.
(31) Liu, S.; Fang, X.; Lu, B.; Yan, D. *Nat. Commun.* **2020**, *11*, 4649.
(32) Bian, L.; Shi, H.; Wang, X.; Ling, K.; Ma, H.; Li, M.; Cheng, Z.; Ma, C.; Cai, S.; Wu, Q.; Gan, N.; Xu, X.; An, Z.; Huang, W. *J. Am. Chem. Soc.* **2018**, *140*, 10734-10739.
(33) Li, Q.; Zhou, M.; Yang, M.; Yang, Q.; Zhang, Z.; Shi, J. *Nat. Commun.* **2018**, *9*, 734.
