# Supplementary material for: Multicolor ultralong phosphorescence from perovskite-like octahedral α-AlF3
Source: Nat Commun. 2022 Sep 29;13:5712. doi: 10.1038/s41467-022-33540-1 (PMC9522726; doi:10.1038/s41467-022-33540-1)
Supplement: Supplementary file 3 — Description of Additional Supplementary Files [file 41467_2022_33540_MOESM3_ESM.pdf]

File name: **Supplementary Movie 1**
Description: Afterglow from AlF3-Ex 290nm

File name: **Supplementary Movie 2**
Description: Afterglow from AlF3-Ex 350nm

File name: **Supplementary Movie 3**
Description: Afterglow from AlF3-Ex 390nm

File name: **Supplementary Movie 4**
Description: Afterglow from AlCl3-Ex 280 nm and 350 nm

File name: **Supplementary Movie 5**
Description: Afterglow from Ga2O3-Ex 290 nm

File name: **Supplementary Movie 6**
Description: UV testing paper
